# Updated insights into the impact of adjuvant chemotherapy on recurrence and survival after curative resection of liver or lung metastases in colorectal cancer: a rapid review and meta-analysis

**DOI:** 10.1186/s12957-025-03714-4

**Published:** 2025-02-18

**Authors:** Kyota Tatsuta, Mayu Sakata, Tadahiro Kojima, Eisuke Booka, Kiyotaka Kurachi, Hiroya Takeuchi

**Affiliations:** Department of Surgery, Hamamatsu University School of Medicine, 1-20-1, Handayama, Chuo-ku, Hamamatsu, Shizuoka 431-3192 Japan

**Keywords:** Adjuvant chemotherapy, Colorectal cancer, Liver metastasis, Lung metastasis, Systematic review

## Abstract

**Background:**

Colorectal cancer (CRC) frequently metastasizes to the liver and lungs, leading to poor prognosis. Advances in chemotherapy, minimally invasive surgery, and perioperative care have expanded adjuvant chemotherapy (AC) regimens and eligibility for AC. However, the impact of AC after curative resection of distant metastases on recurrence and prognosis remains uncertain. This study evaluated the role of AC in CRC liver and lung metastases, focusing on cases with curative resection based on the latest studies published in the past five years.

**Methods:**

This systematic review followed PRISMA guidelines. Literature searches of Medline and Cochrane Library (2019–2023) identified studies on AC or observation after curative resection of CRC metastases, reporting outcomes such as overall survival (OS) and disease-free survival (DFS). Data analysis was performed using Review Manager and R software, with results expressed as hazard ratios (HR) and 95% confidence intervals (CI).

**Results:**

Seven studies met the eligibility criteria, including one randomized controlled trial and six retrospective studies, encompassing 1580 patients who underwent curative resection (R0) for CRC metastases. This meta-analysis showed a positive trend in OS for the AC group compared to that for the surgery-alone group (HR 0.86, 95% CI: 0.73–1.01; *p* = 0.06), but the difference was insignificant. AC significantly improved DFS (HR 0.81, 95% CI: 0.66–0.99; *p* = 0.04). Subgroup analysis indicated that AC significantly improved DFS and tended to improve OS for liver metastasis. In contrast, AC did not improve OS in cases of lung metastasis.

**Conclusions:**

This meta-analysis suggests that AC demonstrated significant positive effects on DFS. Moreover, AC could contribute to improvements in OS. These findings, supported by the latest research, reinforce the recommendation of AC as a valuable strategy for improving both recurrence and survival outcomes in patients with curatively resected distant CRC metastases.

**Supplementary Information:**

The online version contains supplementary material available at 10.1186/s12957-025-03714-4.

## Background

Colorectal cancer (CRC) is one of the most prevalent cancers worldwide, along with breast, pulmonary, and prostate cancers [1]. CRC frequently spreads to other organs, with the liver being the most common site of metastasis, followed by the lungs [2, 3]. The prognosis of liver and lung metastases of CRC was initially poor. Recently, significant advancements in effective chemotherapy, expansion of surgical criteria, development of innovative surgical techniques, and improvement in radiation therapy have led to substantial improvements in survival rates [4, 5].

Adjuvant chemotherapy (AC) improves postoperative survival by eliminating micrometastatic deposits in patients with cancer at risk of recurrence [6]. Previous randomized controlled trials (RCTs) have shown that fluorouracil-based AC improves disease-free survival (DFS, 5-year DFS AC group 33.5% vs. Surgery-alone group 26.7%) and recurrence-free survival (RFS, 3-year RFS AC group 38.6% vs. Surgery-alone group 32.3%) after curative resection of CRC liver metastases but has no impact on overall survival (OS) [7, 8]. Additionally, a meta-analysis reported no efficacy of AC for CRC lung metastases, but it included cases of incomplete resection (microscopically, R1, or macroscopically residual, R2), warranting further investigation [9–11].

Fluorouracil-based AC was initially used for treatment; in recent years, more potent regimens, such as oxaliplatin, have been introduced [12]. Moreover, the widespread adoption of minimally invasive surgery, including less invasive approaches to traditionally high-risk procedures such as liver and lung resections, and advancements in perioperative management enabled faster recovery times and improved overall treatment tolerability, thereby broadening the scope of patients who can safely undergo AC [13–16]. With these recent advancements, we hypothesized that the administration of AC after curative resection for liver and lung metastases in CRC may improve recurrence and survival outcomes. Therefore, in this meta-analysis, we aimed to evaluate the impact of AC on liver and lung metastases in CRC, expressly limited to cases with curative resection (R0), based on the latest research published in the past five years (2019–2023).

## Methods

### Literature search methodology

This systematic review adhered to the Preferred Reporting Items for Systematic Reviews and Meta-Analyses (PRISMA) standards [17]. A thorough literature search of articles indexed in the Medline, Cochrane Library databases, and Web of Science was conducted using the following terms: (‘chemotheraphy’ OR ‘adjuvant’ OR ‘postoperat’) AND (‘liver neoplasms’ OR ‘lung neoplasms’ OR ‘metasta’) AND (‘colorectal neoplasms’ OR ‘colon’ OR ‘rectum’ OR ‘cancer’ OR ‘carcinoma’) AND (‘prognosis’ OR ‘mortality’ OR ‘survival analysis’ OR ‘outcome’) AND (2019–2023). The detailed literature search strategy is presented in Supplemental Table 1. This study was registered in PROSPERO (CRD42024570490; https://www.crd.york.ac.uk/PROSPERO/display_record.php?RecordID=570490). The analysis was limited to studies published between 2019 and 2023, as this timeframe was selected to reflect the most recent advancements in chemotherapy, surgical techniques, and perioperative management. A final literature search was performed on December 18, 2024. The PRISMA 2020 checklist is available in Supplemental Table 2.

### Eligibility criteria

**Fig. 1:**
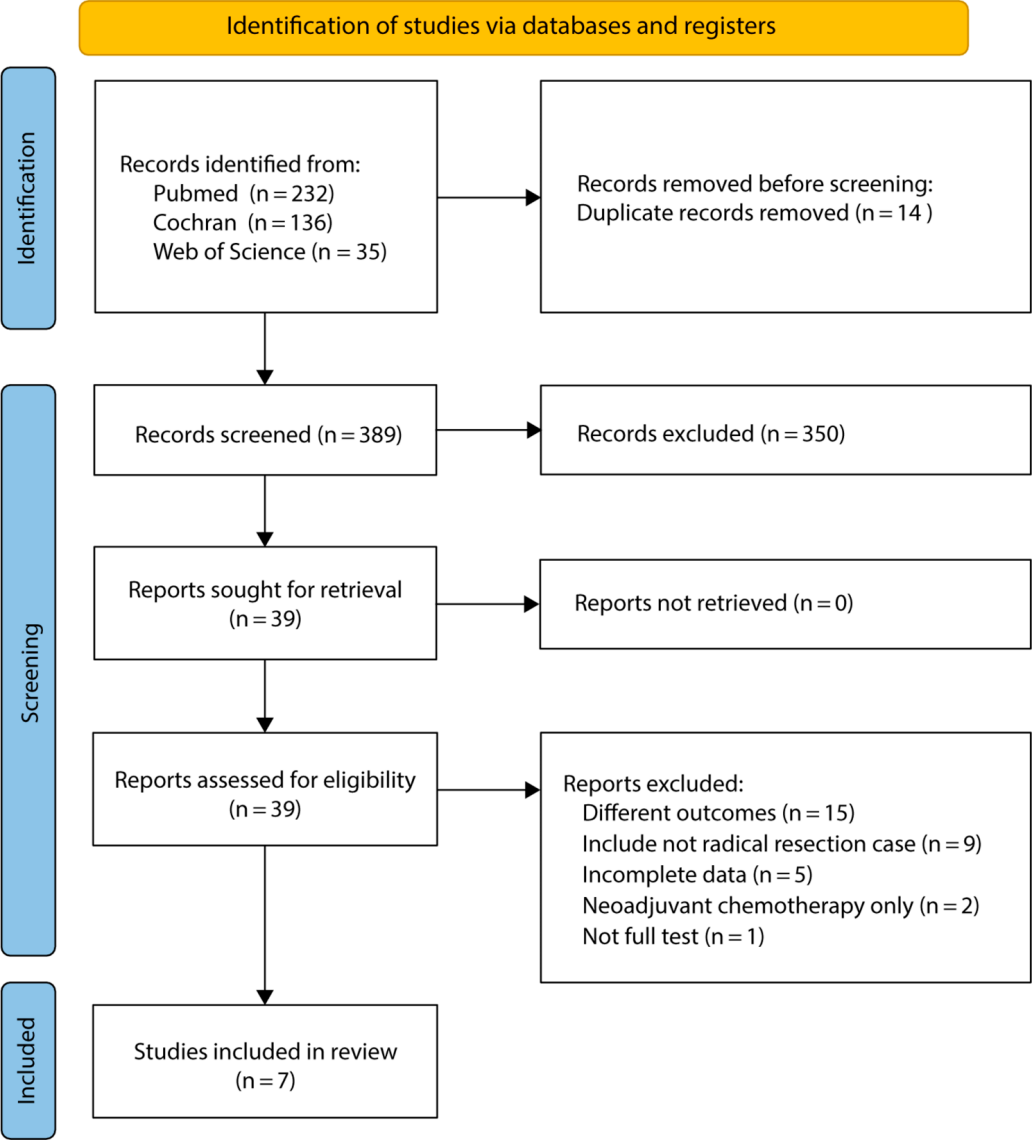
Flow chart of study selection

The search strategy was used to identify relevant studies from the selected databases. Two independent researchers systematically reviewed the studies according to the PRISMA guidelines. After eliminating duplicates, the researchers screened the studies based on titles and abstracts. Only studies that met the predefined inclusion and exclusion criteria were advanced to the next stage, where a thorough review of the full text was conducted. The inclusion criteria were as follows: (1) radical surgery for distant metastasis of CRC; (2) AC or observation after pathological CR; and (3) outcomes including estimate values (hazard ratio [HR] with 95% confidence interval [CI]) for survival, and/or recurrence. The exclusion criteria were as follows: (1) pathological non-curative resection (R1, R2); (2) no desired outcome reported; (3) neoadjuvant chemotherapy only, without AC; and (4) abstracts, meta-analyses, reviews, comments, and letters (Fig. 1).

### Data extraction

Two reviewers independently screened potential abstracts and full texts based on the inclusion criteria. The two reviewers extracted all data from the eligible studies to maintain data consistency and integrity. Any discrepancies were resolved through discussions with a third independent reviewer.

The extracted information included general information such as author names, publication date, source of data, and study period. Essential clinical characteristics such as age, sex, metastatic site, timing of metastasis, AC regimen, location of the primary colorectal cancer, presence or absence of preoperative chemotherapy, and follow-up duration were also recorded.

### Quality and bias assessment

The two reviewers assessed the risk of bias and quality. The risk of bias in non-randomized studies of interventions (ROBINS-I) tool [18] and revised Cochrane risk-of-bias tool for randomized trials (ROB2) [19] were used to assess the risk of bias in the retrospective studies and RCT included in this study, respectively. Funnel plots were used to evaluate publication bias. The Grades of Recommendation, Assessment, Development and Evaluation Working Group (GRADE) framework was employed to assess the level of evidence [20].

### Outcome measures

The primary outcome was OS, and the secondary outcome was DFS. This study integrated RFS into DFS for analysis, treating them as equivalent to evaluating the time to disease recurrence. To assess the impact of AC on long-term outcomes in detail, a subgroup analysis was carried out on the effects of the presence or absence of neoadjuvant chemotherapy and metastatic site (liver or lung). Furthermore, we assessed the significance of the study design (RCT or retrospective study).

### Statistical analysis

Analyses were performed using Review Manager version 5.4 (The Cochrane Collaboration, Oxford, UK) and R software (version 4.4.1, R Foundation for Statistical Computing). Pooled analysis was performed using the Mantel–Haenszel model, and the values are reported as HR with 95% CI. The Z test was used to determine the significance of pooled HR. Begg’s test quantitatively assessed publication bias. *p* < 0.050 was considered statistically significant.

Statistical heterogeneity for each pooled estimate was evaluated using Cochran’s χ2 test and quantified using the I^2^ statistic. In this study, we used a random-effects model to assess the results, as the meta-analysis was small in scale, and the patient characteristics were not adequately matched.

## Results

### Characteristics of included studies

In total, 403 studies were identified using the search strategy. A total of 389 studies were identified after manually removing duplicates, and seven full-text studies met the eligibility criteria for inclusion after the final assessment [21–27]. The PRISMA flow diagram shows the selection strategy and procedure (Fig. 1).

**Fig. 2:**
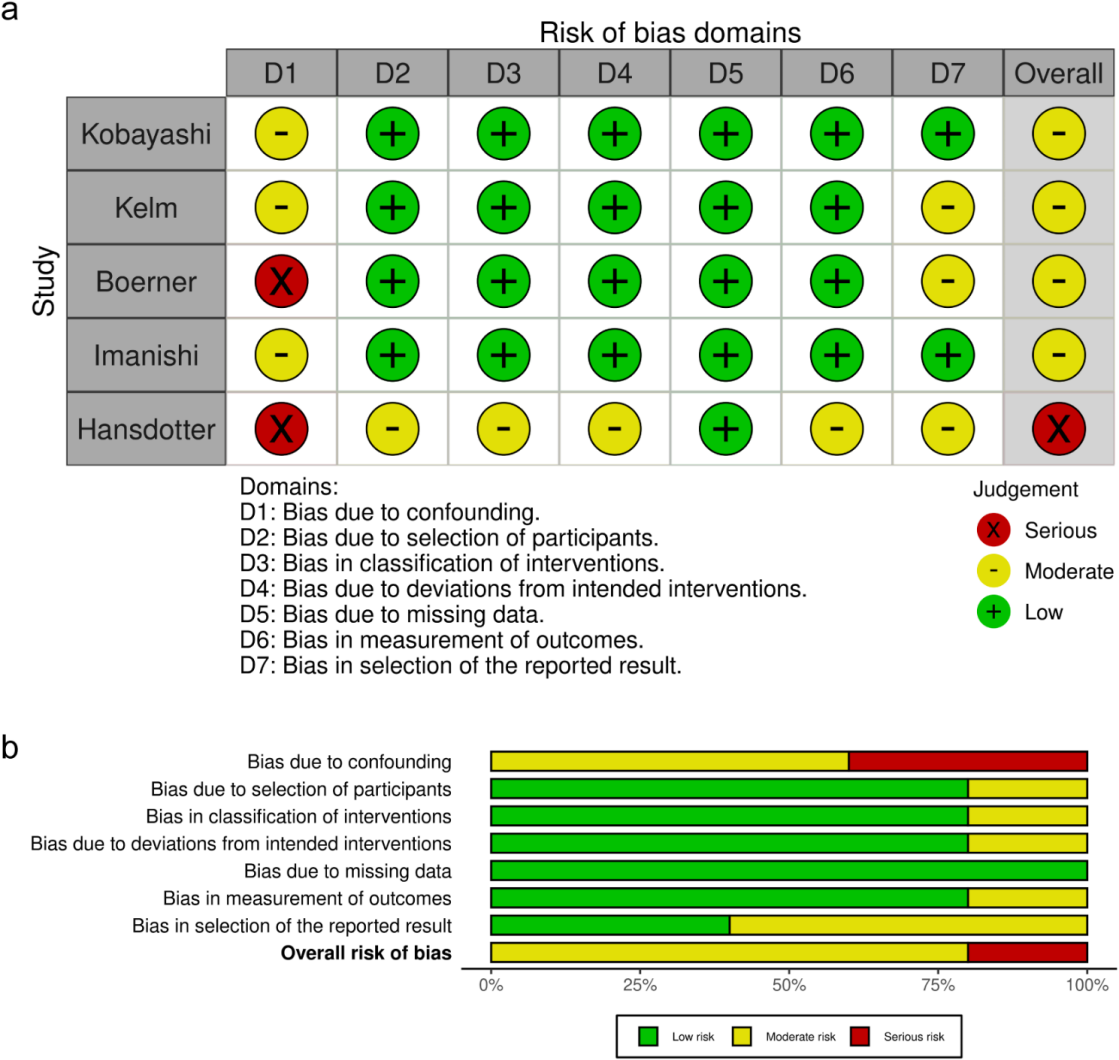
Evaluation of the risk of bias utilizing the ROBINS-I tool for cohort studies (**a**) Risk of bias summary for each included study, (**b**) Risk of bias graph for the included studies ROBINS-I, risk of bias in non-randomized studies of interventions

**Fig. 3:**
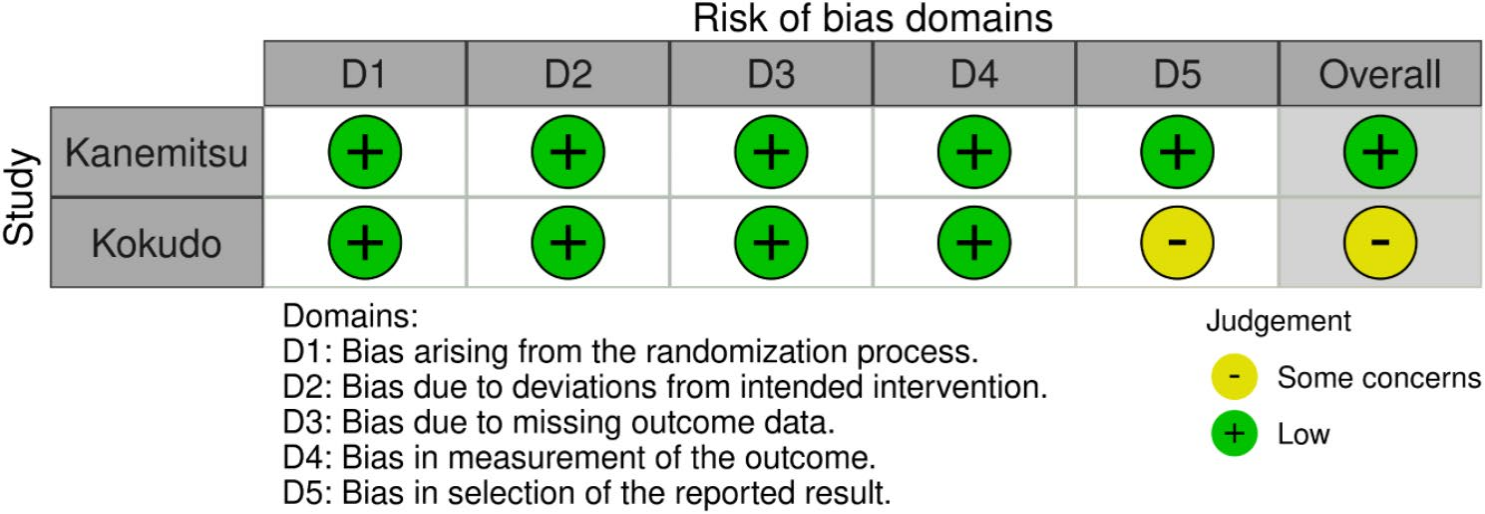
Evaluation of the risk of bias using the ROB2 for randomized studies ROB2, risk of bias assessment tool 2.0

**Fig. 4:**
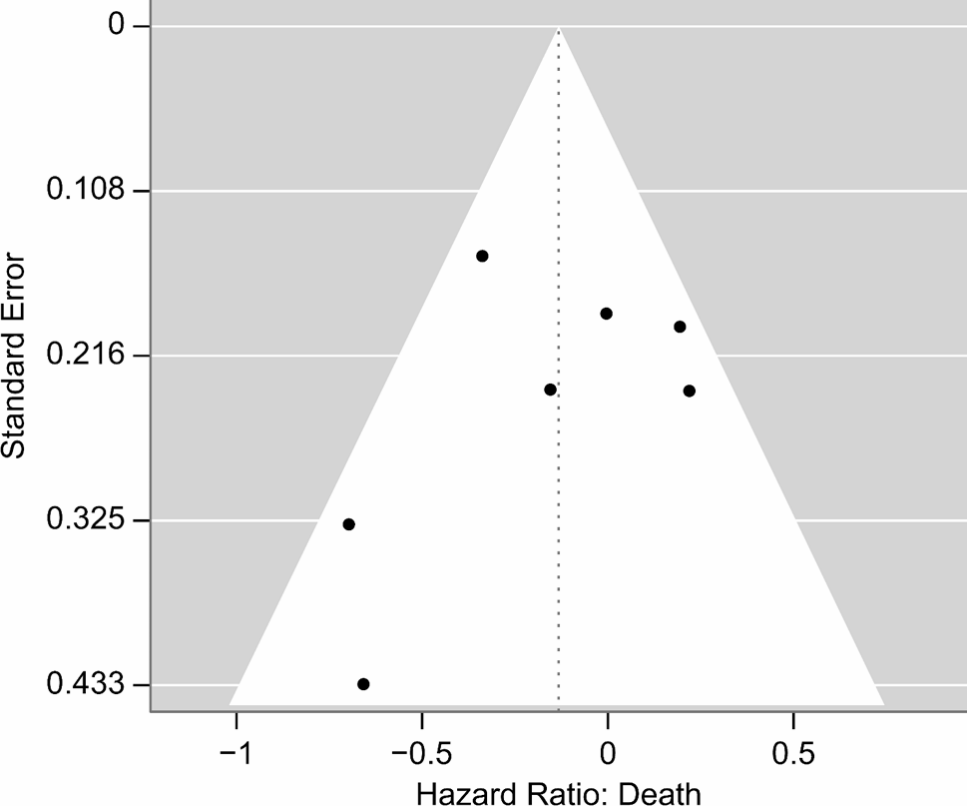
Funnel plot of the included studies

Of the seven studies, two were RCT, and the remaining six were retrospective (Table 1). In addition, the bias of the included studies was graded as low to serious (Figs. 2 and 3, and Supplemental Table 3), and the funnel plots and Begg’s test showed no publication bias (Fig. 4, *p* = 0.77). According to the GRADE framework, the specific details of evidence-level evaluation are shown in Supplemental Table 4, with very low to moderate levels.

**Table 1:** Characteristics of included studies

Study	Year	Country	Study design	Metastasis site	Patients	Comparative outcomes	Follow-up	Age	Sex	Location of the primary colorectal cancer	Timing of metastasis	Preoperative chemotherapy	AC regimen
Kobayashi	2020	Japan	Retrospective	Liver	AC *n* = 211, SA *n* = 211	OS, RFS	79.4 months	AC 63.5 years, SA 65.4 years	Male 268, Female 154	Colon 316, Rectum 106	Synchronous 244 Metachronous 278	No	FOLFOX, FOLFIRI
Kanemitsu	2021	Japan	RCT	Liver	AC *n* = 151, SA *n* = 149	OS, DFS	53.6 months	AC 65 years, SA 63 years	Male 180, Female 120	Colon 232, Rectum 68	Synchronous 167 Metachronous 133	No	mFOLFOX6
Kokudo	2021	Japan	RCT	Liver	AC *n* = 88, SA *n* = 89	OS, RFS	88.3 months	AC 62.3 years, SA 64.4 years	Male 120, Female 57	Colon 110, Rectum 67	Synchronous 79 Metachronous 98	No	UFT/LV
Kelm	2021	Germany	Retrospective	Liver	AC *n* = 34, SA m = 41	OS, DFS	NR	AC 65 years, SA 65 years	Male 48, Female 27	Colon 44, Rectum 31	Metachronous 75	Yes: 13, No: 62	FOLFOX, FOLFIRI, CAPOX, Capecitabine (*n* = 10)
Boerner	2021	USA	Retrospective	Liver	AC *n* = 77, SA *n* = 83	OS, RFS	96 months	AC 59.2 years, SA 67.9 years	Male 87, Female 73	Colon 122, Rectum 38	Metachronous 160	Yes: 21, No: 139	Oxaliplatin or Irinotecan
Imanishi	2019	Japan	Retrospective	Lung	AC m = 192, SA *n* = 192	OS, DFS	54 months	AC 65 years, SA 67 years	Male 212, Female 172	Colon 158, Rectum 226	Synchronous 45 Metachronous 339	No	Fluoropyrimidine (*n* = 136), Oxaliplatin-based, Irinotecan-based
Hansdotter	2023	Sweden	Retrospective	Lung	AC *n* = 29, SA *n* = 33	OS	NR	NR	NR	NR	NR	No	NR

AC, adjuvant chemotherapy; SA, surgery alone; RCT, randomized controlled trial; NR, not reported; USA, united states of America

### Patient characteristics

A total of 1580 patients who underwent curative resection for distant metastases of CRC were included. Among these, 782 and 798 patients were included in the AC and surgery-alone groups, respectively. Distant metastases occurred as metachronous metastasis in 66.9% of cases. A total of 78.7% of patients received oxaliplatin- or irinotecan-based chemotherapy regimens (Table 1). The median observation period was 79.4 months. Two studies included patients who received neoadjuvant chemotherapy [22, 27].

### Primary outcome

**Fig. 5:**
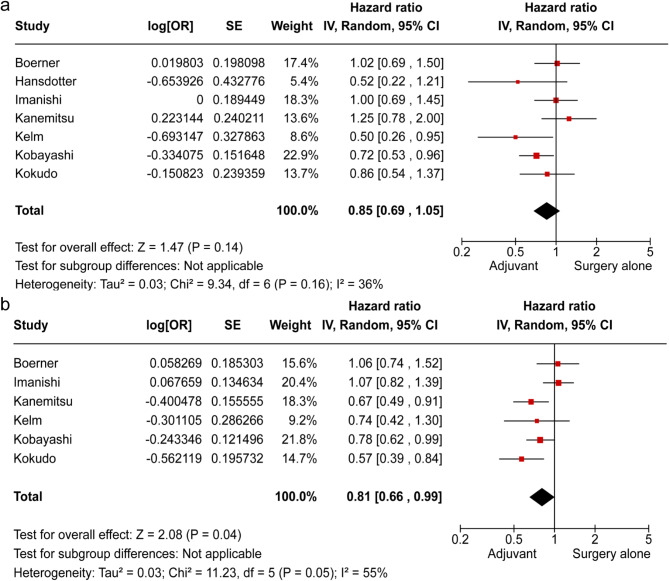
Hazard ratio analysis for adjuvant chemotherapy vs. surgery alone. (**a**) overall survival, (**b**) disease-free survival

OS tended to improve in the AC group compared to the surgery-alone group, but there was no significant difference (HR 0.86, 95% CI: 0.73–1.01; *p* = 0.06; Fig. 5a).

### Secondary outcome

DFS significantly improved in the AC group compared to the surgery-alone group (HR 0.81, 95% CI: 0.66–0.99; *p* = 0.04; Fig. 5b).

### Subgroup analysis

**Fig. 6:**
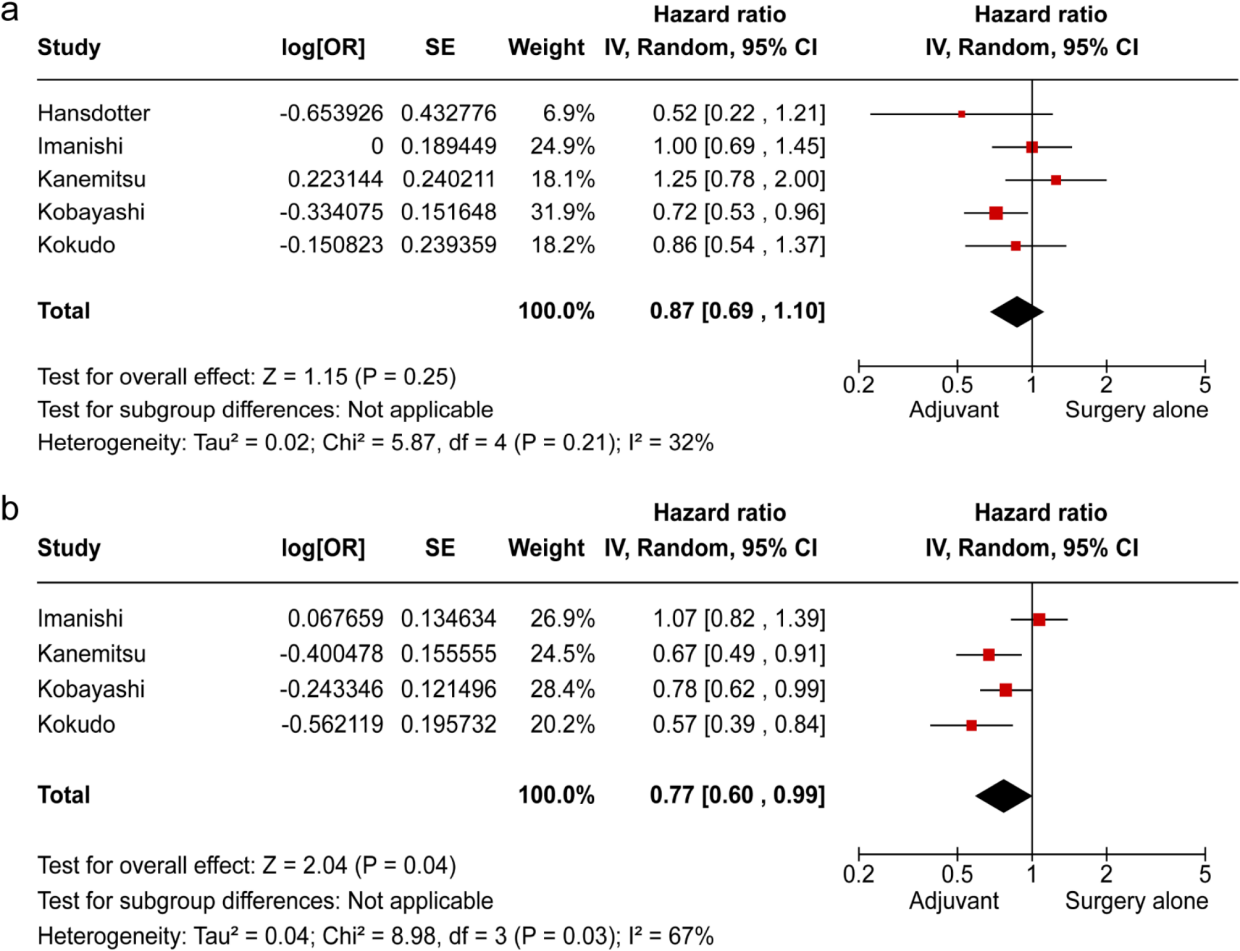
Subgroup analysis for adjuvant chemotherapy vs. surgery alone excluding those who received neoadjuvant chemotherapy (**a**) overall survival, (**b**) disease-free survival

We performed the same analysis, excluding the two studies that included patients who received neoadjuvant chemotherapy [22, 27]. DFS remained significantly improved in the AC group compared with the surgery-alone group (HR 0.77, 95% CI: 0.60–0.99; *p* = 0.03), with no difference in OS between the groups (Fig. 6).

**Fig. 7:**
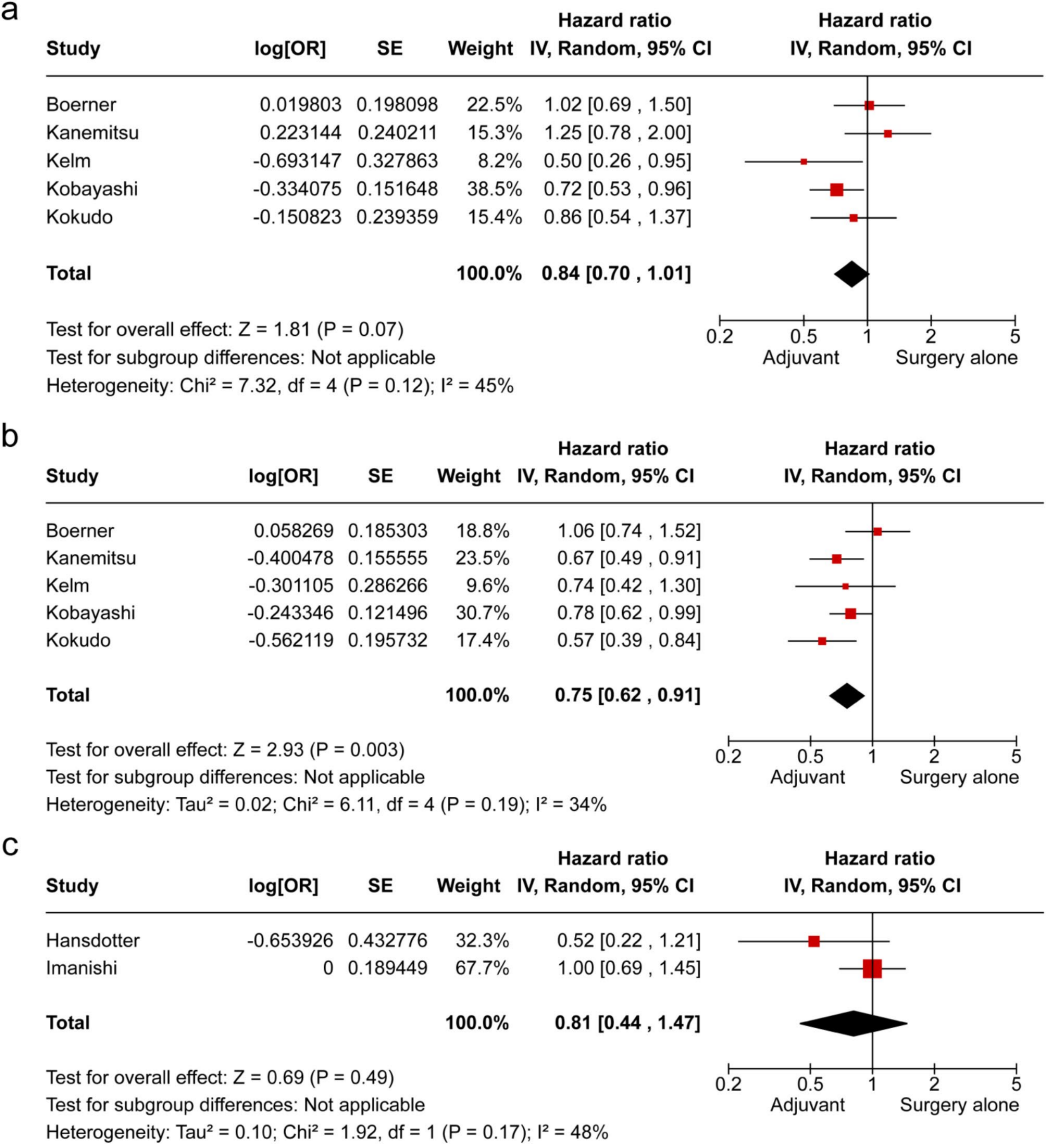
Subgroup analysis for adjuvant chemotherapy vs. surgery alone by metastatic site (**a**, **b**) liver metastasis; (**a**) overall survival, (**b**) disease-free survival, (**c**) lung metastasis; overall survival

The efficacy of AC was further evaluated according to the metastatic organ in CRC. In cases of liver metastasis, DFS significantly improved in the AC group compared with that in the surgery-alone group (HR 0.75, 95% CI: 0.65–0.87), and OS showed a notable trend toward improvement; however, there was no statistically significant difference between the groups (Fig. 7a, b). In contrast, for lung metastases, no improvement in OS was observed in the AC group (Fig. 7c).

**Fig. 8:**
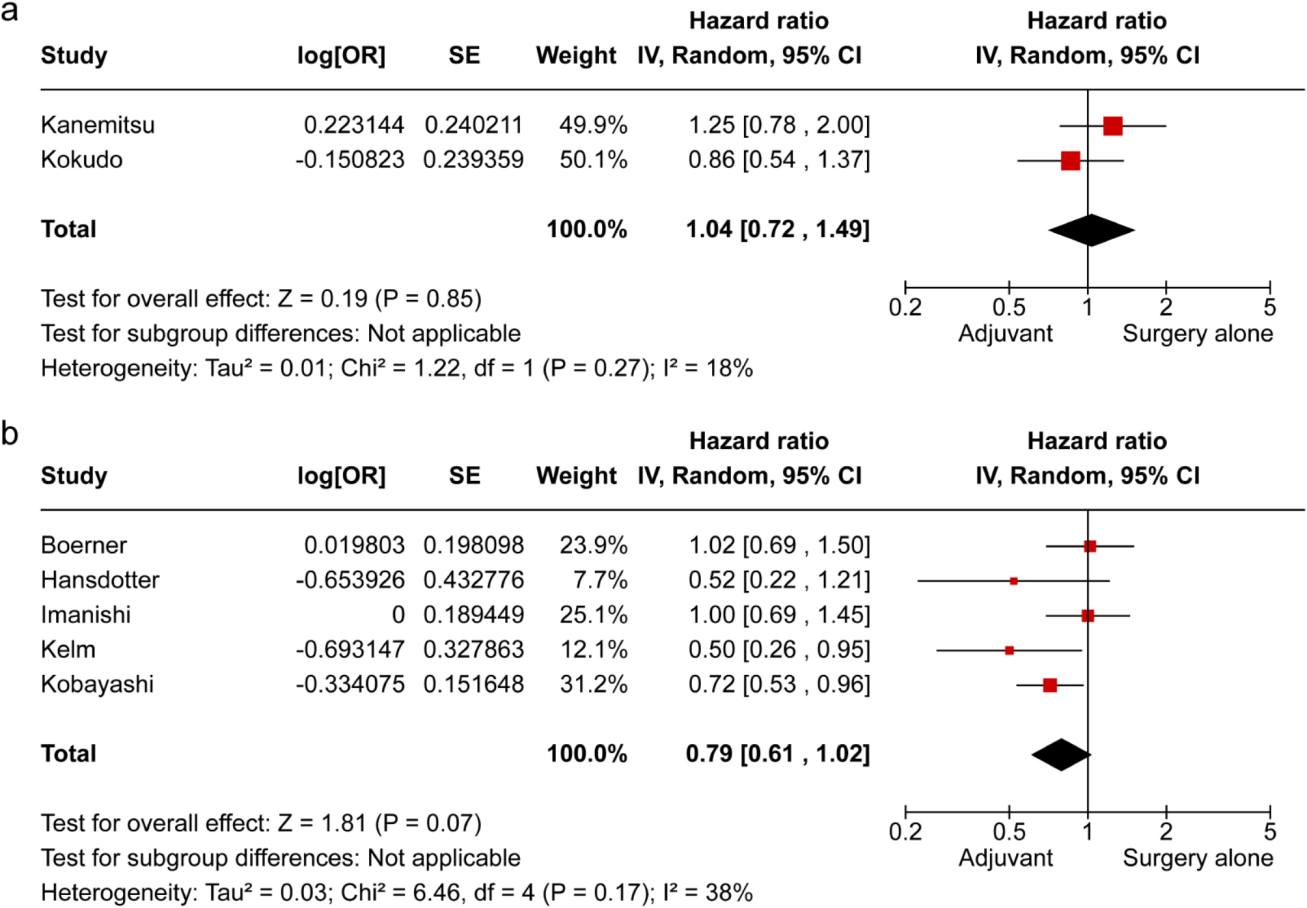
Subgroup analysis of overall survival divided into randomized controlled trials and retrospective studies (**a**) randomized controlled trials, (**b**) retrospective studies

Finally, we evaluated the significance of the study design. The RCT analysis revealed no significant effect of AC for OS (Fig. 8a), while the retrospective study suggested a potential benefit of AC (HR 0.79, 95% CI: 0.61–1.02; *p* = 0.07; Fig. 8b).

## Discussion

In this systematic review and meta-analysis, we assessed the efficacy of AC following curative resection for distant metastases of CRC based on studies published from 2019 onward. Our findings confirmed the advantage of AC in reducing recurrence while also showing a positive trend toward improved OS. The previous meta-analysis of AC after distant metastasis of CRC was based primarily on studies from the 2000s to early 2010s [28], necessitating a re-examination using more recent data. Our results, based on the latest research, further emphasize the benefit of AC for both preventing recurrence and potentially improving survival after curative resection for distant CRC metastases.

Recent understanding of AC has suggested its beneficial role in patients with CRC. For stage III and selected patients with stage II CRC, a fluorouracil-based regimen improved absolute survival by 5–10%, and the oxaliplatin-based regimen reduced the relative risk of recurrence of stage III disease to approximately 30% [29, 30]. Administering AC after resection of distant metastases, including perioperative chemotherapy combined with neoadjuvant chemotherapy, is recommended in various guidelines; however, it is not yet universally accepted as standard treatment [31, 32]. One contributing factor to this recommendation is that AC after resectioning distant metastases is recognized for reducing recurrence but not improving survival [31]. Although our study did not observe a significant difference, we identified a trend suggesting that AC contributed to extending OS, affirming its potential benefit. This result may be attributed to the expansion of AC regimens and enhanced eligibility for AC through advancements in minimally invasive surgery and perioperative management. Restricting the review to studies published in the past five years (2019–2023) allowed for incorporating the most recent advancements in the field.

The current recommendations for AC for curative resection of liver metastasis are based on the results of the EORTC Intergroup Trial 40,983 comparing perioperative FOLFOX4 chemotherapy and surgery versus surgery alone for resectable liver metastases from CRC [33, 34]. Owing to the potential impact of preoperative treatment on outcomes, these results regarding its efficacy should be considered with caution. We conducted a subgroup analysis excluding studies that used perioperative chemotherapy. This analysis showed a significant extension of DFS and the potential to improve OS, confirming its potential benefit of AC alone. Conversely, a National Database study reported differences in the effectiveness of neoadjuvant chemotherapy compared to AC at the time of curative resection for liver or lung metastases [35]. This study found that neoadjuvant chemotherapy demonstrated superior outcomes in non-academic centers, whereas this superiority was not observed in academic centers [35]. A high-quality prospective RCT is essential to determine whether preoperative chemotherapy or AC is superior as the optimal treatment strategy for the resection of distant metastases in CRC.

The subgroup analysis by organ metastasis confirmed the efficacy of AC for liver metastasis, similar to the overall analysis. AC showed extreme efficacy in improving DFS, consistent with the most recent meta-analysis [36]. In contrast, the analysis did not confirm the efficacy of AC for lung metastasis, which aligns with a previous meta-analysis [9]. In general, liver metastases have a poorer prognosis than lung metastases [37, 38], suggesting they may have benefited more from AC. Additionally, the different therapeutic effects may be due to molecular differences, e.g., KRAS mutation, BRAF, and microsatellite instability, depending on the metastatic site [38–40]. We believe selecting AC based on molecular biomarkers is crucial for identifying the most effective treatment strategy. In contrast, a recent meta-analysis revealed that perioperative chemotherapy for lung metastasis reduces recurrence and improves prognosis [39]. Perioperative chemotherapy, not AC alone, may contribute to the improvement of prognosis and recurrence in lung metastasis. Further research is needed to understand the treatment interventions for lung metastases fully.

The lack of a significant difference in OS may be attributed to the treatment strategies employed after recurrence. In CRC with liver metastases, approximately 60–70% of patients experience a recurrence in the remaining liver [41]. Systemic chemotherapy and repeat hepatectomy are common treatment options for such recurrences, and aggressive repeat hepatectomy can improve prognosis [42]. Large-scale retrospective studies have also demonstrated that repeated aggressive hepatectomies positively impact both OS and DFS, regardless of the use of AC [43]. Similarly, repeated pulmonary resections contribute to favorable outcomes in cases of lung metastases [44]. Therefore, these potential improvements from repeated surgical interventions might obscure the specific impact of AC on OS.

This study has several limitations. Most included studies were retrospective, providing very low to moderate-quality evidence. Different results for OS were observed between the RCTs and retrospective studies, with the latter showing positive trends in AC. Therefore, the potential bias inherent in observational study designs warrants careful evaluation. Furthermore, the heterogeneity in chemotherapy regimens and the lack of uniformity in clinical and oncological characteristics—such as the number, size, and timing of metastases—pose challenges to standardization. Addressing these issues is crucial for enabling more accurate evaluations and advancing research into the efficacy of AC following curative resection of distant CRC metastases.

## Conclusions

This meta-analysis highlights the beneficial role of AC in reducing recurrence and suggests a potential improvement in OS following curative resection of distant metastases in CRC. Particularly, AC appears to be effective for liver metastases. These results should be interpreted cautiously due to the high heterogeneity of the patient populations and outcomes in the available studies.

## Electronic supplementary material

Below is the link to the electronic supplementary material.

Supplementary Material 1

## Data Availability

No datasets were generated or analysed during the current study.

## Abbreviations

AC: Adjuvant chemotherapy
CRC: Colorectal cancer
OS: Overall survival
RFS: Recurrence-free survival
DFS: Disease-free survival
ROBINS-I: Risk of bias in non-randomized studies of interventions
ROB2: Risk-of-bias tool for randomized trials
