# Supplementary material for: Updated insights into the impact of adjuvant chemotherapy on recurrence and survival after curative resection of liver or lung metastases in colorectal cancer: a rapid review and meta-analysis
Source: World J Surg Oncol. 2025 Feb 18;23:56. doi: 10.1186/s12957-025-03714-4 (PMC11834510; doi:10.1186/s12957-025-03714-4)
Supplement: Supplementary file 1 — Supplementary Material 1 [file 12957_2025_3714_MOESM1_ESM.docx]

**Supplemental Table 1.** Detailed literature search strategy

| **Medline** | | |
| --- | --- | --- |
| **ID** | **Search** | **Hits** |
| L1 | S CHEMOTHERAPY, ADJUVANT+NT/CT OR ADJUVANT?(3A)(CHEMOTHERAP? OR THERAP?) OR (POSTOPERAT? OR POST(W)OPERAT?)(3A)ADJUVANT? | 117576 |
| L2 | S LIVER NEOPLASMS+NT/CT(L)SECONDARY OR LUNG NEOPLASMS+NT/CT(L)SECONDARY OR (LUNG? OR PULMONARY? OR LIVER? OR HEPATIC?)(3A)METASTA? | 129200 |
| L3 | S COLORECTAL NEOPLASMS+NT/CT | 251783 |
| L4 | S (COLORECT? OR RECTAL? OR COLON?)(3A)(NEOPLASM? OR TUMOR? OR TUMOUR? OR CANCER? OR ADENO? OR ?CARCINO?) OR LARGE(1W)(INTESTIN? OR BOWEL)(3A)(NEOPLASM? OR TUMOR? OR TUMOUR? OR CANCER? OR ADENO? OR ?CARCINO?) | 349119 |
| L5 | S PROGNOSIS+NT/CT OR PROGNOS? | 2555846 |
| L6 | S MORTALITY+NT/CT OR SURVIVAL ANALYSIS+NT/CT OR MORTALIT? OR SURVIV? OR OUTCOME? | 5565530 |
| L7 | S L1 AND L2 AND (L3 OR L4) AND (L5 OR L6) | 1886 |
| L8 | S L7 AND EN/LA AND (2019-2023)/PY | 274 |
| L9 | S L8 NOT (CASE REPORT?/DT OR CASE(W)REPORT?/TI) | 232 |
| **Cochrane** | | |
| **ID** | **Search** | **Hits** |
| #1 | [mh "CHEMOTHERAPY, ADJUVANT"] OR (ADJUVANT* NEAR/3 (CHEMOTHERAP* OR THERAP*) OR (POSTOPERAT* OR POST-OPERAT*) NEAR/3 ADJUVANT*):TI,KW,AB | 200506 |
| #2 | [mh "LIVER NEOPLASMS"/SC] OR [mh "LUNG NEOPLASMS"/SC] OR ((LUNG* OR PULMONARY* OR LIVER* OR HEPATIC*) NEAR/3 METASTA*):TI,KW,AB | 6399 |
| #3 | [mh "Colorectal Neoplasms"] | 13061 |
| #4 | (Colorect* or rectal* or COLON*) near/3 (NEOPLASM* or TUMOR* or TUMOUR* or CANCER* or adeno* or carcino*) or large next (intestin* or BOWEL*) near/3 (NEOPLASM* or TUMOR* or TUMOUR* or CANCER* or adeno* or carcino*) | 30604 |
| #5 | [mh MORTALITY] OR [mh "SURVIVAL ANALYSIS"] OR (MORTALIT* OR SURVIV* OR OUTCOME*):TI,KW,AB | 986317 |
| #6 | [mh PROGNOSIS] OR PROGNOS*:TI,KW,AB | 263914 |
| #7 | #1 AND #2 AND (#3 OR #4) AND (#5 OR #6) | 523 |
| #8 | #7 with Cochrane Library publication date Between Jan 2019 and Dec 2023, in Cochrane Reviews, Cochrane Protocols | 2 |
| #9 | #7 with Publication Year from 2019 to 2023, in Trials | 136 |
| **Web of Science** | | |
| **ID** | **Search** | **Hits** |
| #1 | colorectal cancer (All Fields) and liver (All Fields) and lung (All Fields) and metastasis (All Fields) and adjuvant (All Fields) and chemotherapy (All Fields) | 132 |
| #2 | #1 with publication date (2019-01-01 to 2023-12-31) | 35 |

**Supplemental Table 2. PRISMA Checklist**

| **Section and Topic** | **Item #** | **Checklist item** | **Location where item is reported** |
| --- | --- | --- | --- |
| **TITLE** | | |  |
| Title | 1 | Identify the report as a systematic review. | P1 |
| **ABSTRACT** | | |  |
| Abstract | 2 | See the PRISMA 2020 for Abstracts checklist. | P2-3 |
| **INTRODUCTION** | | |  |
| Rationale | 3 | Describe the rationale for the review in the context of existing knowledge. | P4 |
| Objectives | 4 | Provide an explicit statement of the objective(s) or question(s) the review addresses. | P5 |
| **METHODS** | | |  |
| Eligibility criteria | 5 | Specify the inclusion and exclusion criteria for the review and how studies were grouped for the syntheses. | P6 |
| Information sources | 6 | Specify all databases, registers, websites, organisations, reference lists and other sources searched or consulted to identify studies. Specify the date when each source was last searched or consulted. | P5 |
| Search strategy | 7 | Present the full search strategies for all databases, registers and websites, including any filters and limits used. | P5 |
| Selection process | 8 | Specify the methods used to decide whether a study met the inclusion criteria of the review, including how many reviewers screened each record and each report retrieved, whether they worked independently, and if applicable, details of automation tools used in the process. | P6 |
| Data collection process | 9 | Specify the methods used to collect data from reports, including how many reviewers collected data from each report, whether they worked independently, any processes for obtaining or confirming data from study investigators, and if applicable, details of automation tools used in the process. | P5-6 |
| Data items | 10a | List and define all outcomes for which data were sought. Specify whether all results that were compatible with each outcome domain in each study were sought (e.g. for all measures, time points, analyses), and if not, the methods used to decide which results to collect. | P6-8 |
|  | 10b | List and define all other variables for which data were sought (e.g. participant and intervention characteristics, funding sources). Describe any assumptions made about any missing or unclear information. | P6-8 |
| Study risk of bias assessment | 11 | Specify the methods used to assess risk of bias in the included studies, including details of the tool(s) used, how many reviewers assessed each study and whether they worked independently, and if applicable, details of automation tools used in the process. | P7 |
| Effect measures | 12 | Specify for each outcome the effect measure(s) (e.g. risk ratio, mean difference) used in the synthesis or presentation of results. | P7-8 |
| Synthesis methods | 13a | Describe the processes used to decide which studies were eligible for each synthesis (e.g. tabulating the study intervention characteristics and comparing against the planned groups for each synthesis (item #5)). | P5-7 |
|  | 13b | Describe any methods required to prepare the data for presentation or synthesis, such as handling of missing summary statistics, or data conversions. | P5-7 |
|  | 13c | Describe any methods used to tabulate or visually display results of individual studies and syntheses. | P5-7 |
|  | 13d | Describe any methods used to synthesize results and provide a rationale for the choice(s). If meta-analysis was performed, describe the model(s), method(s) to identify the presence and extent of statistical heterogeneity, and software package(s) used. | P5-7 |
|  | 13e | Describe any methods used to explore possible causes of heterogeneity among study results (e.g. subgroup analysis, meta-regression). | P6-7 |
|  | 13f | Describe any sensitivity analyses conducted to assess robustness of the synthesized results. | We did not perform a sensitive analysis. |
| Reporting bias assessment | 14 | Describe any methods used to assess risk of bias due to missing results in a synthesis (arising from reporting biases). | P7 |
| Certainty assessment | 15 | Describe any methods used to assess certainty (or confidence) in the body of evidence for an outcome. | P7 |
| **RESULTS** | | |  |
| Study selection | 16a | Describe the results of the search and selection process, from the number of records identified in the search to the number of studies included in the review, ideally using a flow diagram. | P8, Figure1 |
|  | 16b | Cite studies that might appear to meet the inclusion criteria, but which were excluded, and explain why they were excluded. | Figure1 |
| Study characteristics | 17 | Cite each included study and present its characteristics. | P8, Table1 |
| Risk of bias in studies | 18 | Present assessments of risk of bias for each included study. | Figure2,3 |
| Results of individual studies | 19 | For all outcomes, present, for each study: (a) summary statistics for each group (where appropriate) and (b) an effect estimate and its precision (e.g. confidence/credible interval), ideally using structured tables or plots. | P9, Figure5-7 |
| Results of syntheses | 20a | For each synthesis, briefly summarise the characteristics and risk of bias among contributing studies. | P8, Figure2,3 |
|  | 20b | Present results of all statistical syntheses conducted. If meta-analysis was done, present for each the summary estimate and its precision (e.g. confidence/credible interval) and measures of statistical heterogeneity. If comparing groups, describe the direction of the effect. | P9, Figure5-7 |
|  | 20c | Present results of all investigations of possible causes of heterogeneity among study results. | P8 |
|  | 20d | Present results of all sensitivity analyses conducted to assess the robustness of the synthesized results. | We did not perform a sensitive analysis. |
| Reporting biases | 21 | Present assessments of risk of bias due to missing results (arising from reporting biases) for each synthesis assessed. | P9, Figure2,3 |
| Certainty of evidence | 22 | Present assessments of certainty (or confidence) in the body of evidence for each outcome assessed. | Supplemental Table4 |
| **DISCUSSION** | | |  |
| Discussion | 23a | Provide a general interpretation of the results in the context of other evidence. | P10-12 |
|  | 23b | Discuss any limitations of the evidence included in the review. | P12,13 |
|  | 23c | Discuss any limitations of the review processes used. | P12,13 |
|  | 23d | Discuss implications of the results for practice, policy, and future research. | P10-13 |
| **OTHER INFORMATION** | | |  |
| Registration and protocol | 24a | Provide registration information for the review, including register name and registration number, or state that the review was not registered. | P5 |
|  | 24b | Indicate where the review protocol can be accessed, or state that a protocol was not prepared. | P14 |
|  | 24c | Describe and explain any amendments to information provided at registration or in the protocol. | Supplemental Table1, 3, 4 |
| Support | 25 | Describe sources of financial or non-financial support for the review, and the role of the funders or sponsors in the review. | P13 |
| Competing interests | 26 | Declare any competing interests of review authors. | P13 |
| Availability of data, code and other materials | 27 | Report which of the following are publicly available and where they can be found: template data collection forms; data extracted from included studies; data used for all analyses; analytic code; any other materials used in the review. | Figure1-7, Supplemental Table1-4 |

**Supplemental Table 3. Detailed Evaluation of the risk of bias utilizing the ROBINS-I tool for cohort studies**

## Specify the review question

| Participants | Patients with metastasized colorectal cancer who underwent curative resection |
| --- | --- |
| Experimental intervention | Adjuvant chemotherapy |
| Comparator | Surgery alone |
| Outcomes | OS, DFS |

## List the confounding domains relevant to all or most studies

| Not applicable (NA) |
| --- |

## List co-interventions that could be different between intervention groups and that could impact on outcomes

| Neoadjuvant chemotherapy |
| --- |

# ROBINS-I tool (Stage II): Kobayashi^23^, doi: 10.1007/s11605-019-04250-9.

## Specify a target randomized trial specific to the study

| Design | Matched |
| --- | --- |
| Participants | Patients with liver metastasized colorectal cancer who underwent curative resection |
| Experimental intervention | Adjuvant chemotherapy |
| Comparator | Surgery alone |

## Is your aim for this study…?

| ■ | to assess the effect of *assignment to* intervention |
| --- | --- |
| □ | to assess the effect of *starting and adhering to* intervention |

## Specify the outcome

| OS, RFS |
| --- |

## Specify the numerical result being assessed

| OS Adjuvant chemotherapy vs Surgery alone, HR 0.716, 95%CI 0.532-0.964  RFS Adjuvant chemotherapy vs Surgery alone, HR 0.784, 95%CI 0.618-0.995 |
| --- |

## Preliminary consideration of confounders

| **(i) Confounding domains listed in the review protocol** | | | | |
| --- | --- | --- | --- | --- |
| Confounding domain | Measured variable(s) | Is there evidence that controlling for this variable was unnecessary?* | Is the confounding domain measured validly and reliably by this variable (or these variables)? | OPTIONAL: Is failure to adjust for this variable (alone) expected to favour the experimental intervention or the comparator? |
| Age | Clinical data | No | Yes | Unpredictable |
| Timing of liver metastasis | Clinical data | No | Yes | Unpredictable |
| Preoperative chemotherapy | Clinical data | No | Yes | Unpredictable |
| AC regimen | Clinical data | No | Yes | Unpredictable |

| **(ii) Additional confounding domains relevant to the setting of this particular study, or which the study authors identified as important** | | | | |
| --- | --- | --- | --- | --- |
| Confounding domain | Measured variable(s) | Is there evidence that controlling for this variable was unnecessary?* | Is the confounding domain measured validly and reliably by this variable (or these variables)? | OPTIONAL: Is failure to adjust for this variable (alone) expected to favour the experimental intervention or the comparator? |
| NA |  |  |  |  |

* In the context of a particular study, variables can be demonstrated not to be confounders and so not included in the analysis: (a) if they are not predictive of the outcome; (b) if they are not predictive of intervention; or (c) because adjustment makes no or minimal difference to the estimated effect of the primary parameter. Note that “no statistically significant association” is not the same as “not predictive”.

## Preliminary consideration of co-interventions

| **(i) Co-interventions listed in the review protocol** | | |
| --- | --- | --- |
| Co-intervention | Is there evidence that controlling for this co-intervention was unnecessary (e.g. because it was not administered)? | Is presence of this co-intervention likely to favour outcomes in the experimental intervention or the comparator |
| NA |  |  |

| **(ii) Additional co-interventions relevant to the setting of this particular study, or which the study authors identified as important** | | |
| --- | --- | --- |
| Co-intervention | Is there evidence that controlling for this co-intervention was unnecessary (e.g. because it was not administered)? | Is presence of this co-intervention likely to favour outcomes in the experimental intervention or the comparator |
| NA |  |  |

## Risk of bias assessment

|  | **Signalling questions** | **Description** | **Response options** |
| --- | --- | --- | --- |
| **Bias due to confounding** | | | |
|  | 1.1 Is there potential for confounding of the effect of intervention in this study?  **If N/PN to 1.1:** the study can be considered to be at low risk of bias due to confounding and no further signalling questions need be considered |  | Y |
|  | **If Y/PY to 1.1**: determine whether there is a need to assess time-varying confounding: |  |  |
|  | 1.2. Was the analysis based on splitting participants’ follow up time according to intervention received?  **If N/PN**, answer questions relating to baseline confounding (1.4 to 1.6)  **If Y/PY**, go to question 1.3. |  | N |
|  | 1.3. Were intervention discontinuations or switches likely to be related to factors that are prognostic for the outcome?  **If N/PN**, answer questions relating to baseline confounding (1.4 to 1.6)  **If Y/PY**, answer questions relating to both baseline and time-varying confounding (1.7 and 1.8) |  | NA |

|  | **Questions relating to baseline confounding only** | | |
| --- | --- | --- | --- |
|  | 1.4. Did the authors use an appropriate analysis method that controlled for all the important confounding domains? | The authors conducted a propensity score matching analysis. But, there remain several confounding factors, including variations in treatment outcomes across facilities and differences in the implementation systems for adjuvant chemotherapy. | N |
|  | 1.5. **If Y/PY to 1.4**: Were confounding domains that were controlled for measured validly and reliably by the variables available in this study? |  | NA |
|  | 1.6. Did the authors control for any post-intervention variables that could have been affected by the intervention? |  | NA |
|  | **Questions relating to baseline and time-varying confounding** | |  |
|  | 1.7. Did the authors use an appropriate analysis method that controlled for all the important confounding domains and for time-varying confounding? |  | NA |
|  | 1.8. **If Y/PY to 1.7**: Were confounding domains that were controlled for measured validly and reliably by the variables available in this study? |  | NA |
|  | **Risk of bias judgement** |  | Moderate |
|  | Optional: What is the predicted direction of bias due to confounding? |  | Favours comparator |

| **Bias in selection of participants into the study** | | | |
| --- | --- | --- | --- |
|  | 2.1. Was selection of participants into the study (or into the analysis) based on participant characteristics observed after the start of intervention?  **If N/PN to 2.1:** go to 2.4 |  | N |
|  | 2.2. **If Y/PY to 2.1**: Were the post-intervention variables that influenced selection likely to be associated with intervention?  2.3 **If Y/PY to 2.2**: Were the post-intervention variables that influenced selection likely to be influenced by the outcome or a cause of the outcome? |  | NA |
|  | 2.4. Do start of follow-up and start of intervention coincide for most participants? |  | Y |
|  | 2.5. **If Y/PY to 2.2 and 2.3, or N/PN to 2.4**: Were adjustment techniques used that are likely to correct for the presence of selection biases? |  | NA |
|  | **Risk of bias judgement** |  | Low |
|  | Optional: What is the predicted direction of bias due to selection of participants into the study? |  | Unpredictable |

| **Bias in classification of interventions** | | | |
| --- | --- | --- | --- |
|  | 3.1 Were intervention groups clearly defined? |  | Y |
|  | 3.2 Was the information used to define intervention groups recorded at the start of the intervention? |  | Y |
|  | 3.3 Could classification of intervention status have been affected by knowledge of the outcome or risk of the outcome? |  | N |
|  | **Risk of bias judgement** |  | Low |
|  | Optional: What is the predicted direction of bias due to classification of interventions? |  | Unpredictable |

| **Bias due to deviations from intended interventions** | | | |
| --- | --- | --- | --- |
|  | **If your aim for this study is to assess the effect of assignment to intervention, answer questions 4.1 and 4.2** | |  |
|  | 4.1. Were there deviations from the intended intervention beyond what would be expected in usual practice? |  | N |
|  | 4.2. **If Y/PY to 4.1**: Were these deviations from intended intervention unbalanced between groups *and* likely to have affected the outcome? |  | NA |
|  | **If your aim for this study is to assess the effect of starting and adhering to intervention, answer questions 4.3 to 4.6** | |  |
|  | 4.3. Were important co-interventions balanced across intervention groups? |  | Y |
|  | 4.4. Was the intervention implemented successfully for most participants? |  | Y |
|  | 4.5. Did study participants adhere to the assigned intervention regimen? |  | Y |
|  | 4.6. **If N/PN to 4.3, 4.4 or 4.5**: Was an appropriate analysis used to estimate the effect of starting and adhering to the intervention? |  | NA |
|  | **Risk of bias judgement** |  | Low |
|  | Optional: What is the predicted direction of bias due to deviations from the intended interventions? |  | Unpredictable |

| **Bias due to missing data** | | | |
| --- | --- | --- | --- |
|  | 5.1 Were outcome data available for all, or nearly all, participants? |  | Y |
|  | 5.2 Were participants excluded due to missing data on intervention status? |  | N |
|  | 5.3 Were participants excluded due to missing data on other variables needed for the analysis? |  | N |
|  | 5.4 **If PN/N to 5.1, or Y/PY to 5.2 or 5.3**: Are the proportion of participants and reasons for missing data similar across interventions? |  | NA |
|  | 5.5 **If PN/N to 5.1, or Y/PY to 5.2 or 5.3**: Is there evidence that results were robust to the presence of missing data? |  | NA |
|  | **Risk of bias judgement** |  | Low |
|  | Optional: What is the predicted direction of bias due to missing data? |  | Unpredictable |

| **Bias in measurement of outcomes** | | | |
| --- | --- | --- | --- |
|  | 6.1 Could the outcome measure have been influenced by knowledge of the intervention received? |  | N |
|  | 6.2 Were outcome assessors aware of the intervention received by study participants? |  | N |
|  | 6.3 Were the methods of outcome assessment comparable across intervention groups? |  | Y |
|  | 6.4 Were any systematic errors in measurement of the outcome related to intervention received? |  | N |
|  | **Risk of bias judgement** |  | Low |
|  | Optional: What is the predicted direction of bias due to measurement of outcomes? |  | Unpredictable |

| **Bias in selection of the reported result** | | | |
| --- | --- | --- | --- |
|  | Is the reported effect estimate likely to be selected, on the basis of the results, from... |  |  |
|  | 7.1. ... multiple outcome *measurements* within the outcome domain? |  | N |
|  | 7.2 ... multiple *analyses* of the intervention-outcome relationship? |  | N |
|  | 7.3 ... different *subgroups*? |  | N |
|  | **Risk of bias judgement** |  | Low |
|  | Optional: What is the predicted direction of bias due to selection of the reported result? |  | Unpredictable |

| **Overall bias** | | | |
| --- | --- | --- | --- |
|  | **Risk of bias judgement** |  | Low |
|  | Optional: What is the overall predicted direction of bias for this outcome? |  | Favours comparator |

**ROBINS-I tool (Stage II): Kelm^22^, doi: 10.1186/s12885-021-07941-2**

**Specify a target randomized trial specific to the study**

| Design | Cohort |
| --- | --- |
| Participants | Patients with liver metastasized colorectal cancer who underwent curative resection |
| Experimental intervention | Adjuvant chemotherapy |
| Comparator | Surgery alone |

**Is your aim for this study…?**

| ■ | to assess the effect of *assignment to* intervention |
| --- | --- |
| □ | to assess the effect of *starting and adhering to* intervention |

**Specify the outcome**

| OS, RFS |
| --- |

**Specify the numerical result being assessed**

| OS Adjuvant chemotherapy vs Surgery alone, HR 0.50, 95%CI 0.26-0.94  DFS Adjuvant chemotherapy vs Surgery alone, HR 0.74, 95%CI 0.42-1.29 |
| --- |

**Preliminary consideration of confounders**

| **(i) Confounding domains listed in the review protocol** | | | | |
| --- | --- | --- | --- | --- |
| Confounding domain | Measured variable(s) | Is there evidence that controlling for this variable was unnecessary?* | Is the confounding domain measured validly and reliably by this variable (or these variables)? | OPTIONAL: Is failure to adjust for this variable (alone) expected to favour the experimental intervention or the comparator? |
| Age | Clinical data | No | Yes | Unpredictable |
| Timing of liver metastasis | Clinical data | No | Yes | Unpredictable |
| Preoperative chemotherapy | Clinical data | No | Yes | Unpredictable |
| AC regimen | Clinical data | No | Yes | Unpredictable |

| **(ii) Additional confounding domains relevant to the setting of this particular study, or which the study authors identified as important** | | | | |
| --- | --- | --- | --- | --- |
| Confounding domain | Measured variable(s) | Is there evidence that controlling for this variable was unnecessary?* | Is the confounding domain measured validly and reliably by this variable (or these variables)? | OPTIONAL: Is failure to adjust for this variable (alone) expected to favour the experimental intervention or the comparator? |
| Preoperative chemotherapy | Clinical data | No | Yes | Unpredictable |

* In the context of a particular study, variables can be demonstrated not to be confounders and so not included in the analysis: (a) if they are not predictive of the outcome; (b) if they are not predictive of intervention; or (c) because adjustment makes no or minimal difference to the estimated effect of the primary parameter. Note that “no statistically significant association” is not the same as “not predictive”.

**Preliminary consideration of co-interventions**

| **(i) Co-interventions listed in the review protocol** | | |
| --- | --- | --- |
| Co-intervention | Is there evidence that controlling for this co-intervention was unnecessary (e.g. because it was not administered)? | Is presence of this co-intervention likely to favour outcomes in the experimental intervention or the comparator |
| NA |  |  |

| **(ii) Additional co-interventions relevant to the setting of this particular study, or which the study authors identified as important** | | |
| --- | --- | --- |
| Co-intervention | Is there evidence that controlling for this co-intervention was unnecessary (e.g. because it was not administered)? | Is presence of this co-intervention likely to favour outcomes in the experimental intervention or the comparator |
| NA |  |  |

**Risk of bias assessment**

|  | **Signalling questions** | **Description** | **Response options** |
| --- | --- | --- | --- |
| **Bias due to confounding** | | | |
|  | 1.1 Is there potential for confounding of the effect of intervention in this study?  **If N/PN to 1.1:** the study can be considered to be at low risk of bias due to confounding and no further signalling questions need be considered |  | Y |
|  | **If Y/PY to 1.1**: determine whether there is a need to assess time-varying confounding: |  |  |
|  | 1.2. Was the analysis based on splitting participants’ follow up time according to intervention received?  **If N/PN**, answer questions relating to baseline confounding (1.4 to 1.6)  **If Y/PY**, go to question 1.3. |  | N |
|  | 1.3. Were intervention discontinuations or switches likely to be related to factors that are prognostic for the outcome?  **If N/PN**, answer questions relating to baseline confounding (1.4 to 1.6)  **If Y/PY**, answer questions relating to both baseline and time-varying confounding (1.7 and 1.8) |  | NA |
|  | **Questions relating to baseline confounding only** | | |
|  | 1.4. Did the authors use an appropriate analysis method that controlled for all the important confounding domains? |  | Y |
|  | 1.5. **If Y/PY to 1.4**: Were confounding domains that were controlled for measured validly and reliably by the variables available in this study? | Although this is a cohort study, there are no differences in the patient characteristics. | N |
|  | 1.6. Did the authors control for any post-intervention variables that could have been affected by the intervention? |  | Y |
|  | **Questions relating to baseline and time-varying confounding** | |  |
|  | 1.7. Did the authors use an appropriate analysis method that controlled for all the important confounding domains and for time-varying confounding? |  | NA |
|  | 1.8. **If Y/PY to 1.7**: Were confounding domains that were controlled for measured validly and reliably by the variables available in this study? |  | NA |
|  | **Risk of bias judgement** |  | Moderate |
|  | Optional: What is the predicted direction of bias due to confounding? |  | Unpredictable |

| **Bias in selection of participants into the study** | | | |
| --- | --- | --- | --- |
|  | 2.1. Was selection of participants into the study (or into the analysis) based on participant characteristics observed after the start of intervention?  **If N/PN to 2.1:** go to 2.4 |  | N |
|  | 2.2. **If Y/PY to 2.1**: Were the post-intervention variables that influenced selection likely to be associated with intervention?  2.3 **If Y/PY to 2.2**: Were the post-intervention variables that influenced selection likely to be influenced by the outcome or a cause of the outcome? |  | NA |
|  | 2.4. Do start of follow-up and start of intervention coincide for most participants? |  | Y |
|  | 2.5. **If Y/PY to 2.2 and 2.3, or N/PN to 2.4**: Were adjustment techniques used that are likely to correct for the presence of selection biases? |  | NA |
|  | **Risk of bias judgement** |  | Low |
|  | Optional: What is the predicted direction of bias due to selection of participants into the study? |  | Unpredictable |

| **Bias in classification of interventions** | | | |
| --- | --- | --- | --- |
|  | 3.1 Were intervention groups clearly defined? |  | Y |
|  | 3.2 Was the information used to define intervention groups recorded at the start of the intervention? |  | Y |
|  | 3.3 Could classification of intervention status have been affected by knowledge of the outcome or risk of the outcome? |  | N |
|  | **Risk of bias judgement** |  | Low |
|  | Optional: What is the predicted direction of bias due to classification of interventions? |  | Unpredictable |

| **Bias due to deviations from intended interventions** | | | |
| --- | --- | --- | --- |
|  | **If your aim for this study is to assess the effect of assignment to intervention, answer questions 4.1 and 4.2** | |  |
|  | 4.1. Were there deviations from the intended intervention beyond what would be expected in usual practice? |  | N |
|  | 4.2. **If Y/PY to 4.1**: Were these deviations from intended intervention unbalanced between groups *and* likely to have affected the outcome? |  | NA |
|  | **If your aim for this study is to assess the effect of starting and adhering to intervention, answer questions 4.3 to 4.6** | |  |
|  | 4.3. Were important co-interventions balanced across intervention groups? |  | Y |
|  | 4.4. Was the intervention implemented successfully for most participants? |  | Y |
|  | 4.5. Did study participants adhere to the assigned intervention regimen? |  | Y |
|  | 4.6. **If N/PN to 4.3, 4.4 or 4.5**: Was an appropriate analysis used to estimate the effect of starting and adhering to the intervention? |  | NA |
|  | **Risk of bias judgement** |  | Low |
|  | Optional: What is the predicted direction of bias due to deviations from the intended interventions? |  | Unpredictable |

| **Bias due to missing data** | | | |
| --- | --- | --- | --- |
|  | 5.1 Were outcome data available for all, or nearly all, participants? |  | Y |
|  | 5.2 Were participants excluded due to missing data on intervention status? |  | N |
|  | 5.3 Were participants excluded due to missing data on other variables needed for the analysis? |  | N |
|  | 5.4 **If PN/N to 5.1, or Y/PY to 5.2 or 5.3**: Are the proportion of participants and reasons for missing data similar across interventions? |  | NA |
|  | 5.5 **If PN/N to 5.1, or Y/PY to 5.2 or 5.3**: Is there evidence that results were robust to the presence of missing data? |  | NA |
|  | **Risk of bias judgement** |  | Low |
|  | Optional: What is the predicted direction of bias due to missing data? |  | Unpredictable |

| **Bias in measurement of outcomes** | | | |
| --- | --- | --- | --- |
|  | 6.1 Could the outcome measure have been influenced by knowledge of the intervention received? |  | N |
|  | 6.2 Were outcome assessors aware of the intervention received by study participants? |  | N |
|  | 6.3 Were the methods of outcome assessment comparable across intervention groups? |  | Y |
|  | 6.4 Were any systematic errors in measurement of the outcome related to intervention received? |  | N |
|  | **Risk of bias judgement** |  | Low |
|  | Optional: What is the predicted direction of bias due to measurement of outcomes? |  | Unpredictable |

| **Bias in selection of the reported result** | | | |
| --- | --- | --- | --- |
|  | Is the reported effect estimate likely to be selected, on the basis of the results, from... |  |  |
|  | 7.1. ... multiple outcome *measurements* within the outcome domain? |  | Y |
|  | 7.2 ... multiple *analyses* of the intervention-outcome relationship? |  | N |
|  | 7.3 ... different *subgroups*? |  | N |
|  | **Risk of bias judgement** |  | Moderate |
|  | Optional: What is the predicted direction of bias due to selection of the reported result? |  | Unpredictable |

| **Overall bias** | | | |
| --- | --- | --- | --- |
|  | **Risk of bias judgement** |  | Moderate |
|  | Optional: What is the overall predicted direction of bias for this outcome? |  | Unpredictable |

**ROBINS-I tool (Stage II): Boerner^27^, doi: 10.1016/j.hpb.2020.08.018**

**Specify a target randomized trial specific to the study**

| Design | Cohort |
| --- | --- |
| Participants | Patients with liver metastasized colorectal cancer who underwent curative resection |
| Experimental intervention | Adjuvant chemotherapy |
| Comparator | Surgery alone |

**Is your aim for this study…?**

| ■ | to assess the effect of *assignment to* intervention |
| --- | --- |
| □ | to assess the effect of *starting and adhering to* intervention |

**Specify the outcome**

| OS, RFS |
| --- |

**Specify the numerical result being assessed**

| OS Adjuvant chemotherapy vs Surgery alone, HR 1.02, 95%CI 0.69-1.50  RFS Adjuvant chemotherapy vs Surgery alone, HR 1.06, 95%CI 0.74-1.53 |
| --- |

**Preliminary consideration of confounders**

| **(i) Confounding domains listed in the review protocol** | | | | |
| --- | --- | --- | --- | --- |
| Confounding domain | Measured variable(s) | Is there evidence that controlling for this variable was unnecessary?* | Is the confounding domain measured validly and reliably by this variable (or these variables)? | OPTIONAL: Is failure to adjust for this variable (alone) expected to favour the experimental intervention or the comparator? |
| Age | Clinical data | No | Yes | Favour experimental |
| Timing of liver metastasis | Clinical data | No | Yes | Unpredictable |
| Preoperative chemotherapy | Clinical data | No | Yes | Unpredictable |
| AC regimen | Clinical data | No | Yes | Unpredictable |

| **(ii) Additional confounding domains relevant to the setting of this particular study, or which the study authors identified as important** | | | | |
| --- | --- | --- | --- | --- |
| Confounding domain | Measured variable(s) | Is there evidence that controlling for this variable was unnecessary?* | Is the confounding domain measured validly and reliably by this variable (or these variables)? | OPTIONAL: Is failure to adjust for this variable (alone) expected to favour the experimental intervention or the comparator? |
| Preoperative chemotherapy | Clinical data | No | Yes | Unpredictable |

* In the context of a particular study, variables can be demonstrated not to be confounders and so not included in the analysis: (a) if they are not predictive of the outcome; (b) if they are not predictive of intervention; or (c) because adjustment makes no or minimal difference to the estimated effect of the primary parameter. Note that “no statistically significant association” is not the same as “not predictive”.

**Preliminary consideration of co-interventions**

| **(i) Co-interventions listed in the review protocol** | | |
| --- | --- | --- |
| Co-intervention | Is there evidence that controlling for this co-intervention was unnecessary (e.g. because it was not administered)? | Is presence of this co-intervention likely to favour outcomes in the experimental intervention or the comparator |
| NA |  |  |

| **(ii) Additional co-interventions relevant to the setting of this particular study, or which the study authors identified as important** | | |
| --- | --- | --- |
| Co-intervention | Is there evidence that controlling for this co-intervention was unnecessary (e.g. because it was not administered)? | Is presence of this co-intervention likely to favour outcomes in the experimental intervention or the comparator |
| NA |  |  |

**Risk of bias assessment**

|  | **Signalling questions** | **Description** | **Response options** |
| --- | --- | --- | --- |
| **Bias due to confounding** | | | |
|  | 1.1 Is there potential for confounding of the effect of intervention in this study?  **If N/PN to 1.1:** the study can be considered to be at low risk of bias due to confounding and no further signalling questions need be considered |  | Y |
|  | **If Y/PY to 1.1**: determine whether there is a need to assess time-varying confounding: |  |  |
|  | 1.2. Was the analysis based on splitting participants’ follow up time according to intervention received?  **If N/PN**, answer questions relating to baseline confounding (1.4 to 1.6)  **If Y/PY**, go to question 1.3. |  | N |
|  | 1.3. Were intervention discontinuations or switches likely to be related to factors that are prognostic for the outcome?  **If N/PN**, answer questions relating to baseline confounding (1.4 to 1.6)  **If Y/PY**, answer questions relating to both baseline and time-varying confounding (1.7 and 1.8) |  | NA |
|  | **Questions relating to baseline confounding only** | | |
|  | 1.4. Did the authors use an appropriate analysis method that controlled for all the important confounding domains? |  | N |
|  | 1.5. **If Y/PY to 1.4**: Were confounding domains that were controlled for measured validly and reliably by the variables available in this study? |  | N |
|  | 1.6. Did the authors control for any post-intervention variables that could have been affected by the intervention? |  | N |
|  | **Questions relating to baseline and time-varying confounding** | |  |
|  | 1.7. Did the authors use an appropriate analysis method that controlled for all the important confounding domains and for time-varying confounding? |  | NA |
|  | 1.8. **If Y/PY to 1.7**: Were confounding domains that were controlled for measured validly and reliably by the variables available in this study? |  | NA |
|  | **Risk of bias judgement** | Since there were no differences in patient characteristics other than age, we made it ‘serious.’ | Serious |
|  | Optional: What is the predicted direction of bias due to confounding? |  | Unpredictable |

| **Bias in selection of participants into the study** | | | |
| --- | --- | --- | --- |
|  | 2.1. Was selection of participants into the study (or into the analysis) based on participant characteristics observed after the start of intervention?  **If N/PN to 2.1:** go to 2.4 |  | N |
|  | 2.2. **If Y/PY to 2.1**: Were the post-intervention variables that influenced selection likely to be associated with intervention?  2.3 **If Y/PY to 2.2**: Were the post-intervention variables that influenced selection likely to be influenced by the outcome or a cause of the outcome? |  | NA |
|  | 2.4. Do start of follow-up and start of intervention coincide for most participants? |  | Y |
|  | 2.5. **If Y/PY to 2.2 and 2.3, or N/PN to 2.4**: Were adjustment techniques used that are likely to correct for the presence of selection biases? |  | NA |
|  | **Risk of bias judgement** |  | Low |
|  | Optional: What is the predicted direction of bias due to selection of participants into the study? |  | Unpredictable |

| **Bias in classification of interventions** | | | |
| --- | --- | --- | --- |
|  | 3.1 Were intervention groups clearly defined? |  | Y |
|  | 3.2 Was the information used to define intervention groups recorded at the start of the intervention? |  | Y |
|  | 3.3 Could classification of intervention status have been affected by knowledge of the outcome or risk of the outcome? |  | N |
|  | **Risk of bias judgement** |  | Low |
|  | Optional: What is the predicted direction of bias due to classification of interventions? |  | Unpredictable |

| **Bias due to deviations from intended interventions** | | | |
| --- | --- | --- | --- |
|  | **If your aim for this study is to assess the effect of assignment to intervention, answer questions 4.1 and 4.2** | |  |
|  | 4.1. Were there deviations from the intended intervention beyond what would be expected in usual practice? |  | N |
|  | 4.2. **If Y/PY to 4.1**: Were these deviations from intended intervention unbalanced between groups *and* likely to have affected the outcome? |  | NA |
|  | **If your aim for this study is to assess the effect of starting and adhering to intervention, answer questions 4.3 to 4.6** | |  |
|  | 4.3. Were important co-interventions balanced across intervention groups? |  | Y |
|  | 4.4. Was the intervention implemented successfully for most participants? |  | Y |
|  | 4.5. Did study participants adhere to the assigned intervention regimen? |  | Y |
|  | 4.6. **If N/PN to 4.3, 4.4 or 4.5**: Was an appropriate analysis used to estimate the effect of starting and adhering to the intervention? |  | NA |
|  | **Risk of bias judgement** |  | Low |
|  | Optional: What is the predicted direction of bias due to deviations from the intended interventions? |  | Unpredictable |

| **Bias due to missing data** | | | |
| --- | --- | --- | --- |
|  | 5.1 Were outcome data available for all, or nearly all, participants? |  | Y |
|  | 5.2 Were participants excluded due to missing data on intervention status? |  | N |
|  | 5.3 Were participants excluded due to missing data on other variables needed for the analysis? |  | N |
|  | 5.4 **If PN/N to 5.1, or Y/PY to 5.2 or 5.3**: Are the proportion of participants and reasons for missing data similar across interventions? |  | NA |
|  | 5.5 **If PN/N to 5.1, or Y/PY to 5.2 or 5.3**: Is there evidence that results were robust to the presence of missing data? |  | NA |
|  | **Risk of bias judgement** |  | Low |
|  | Optional: What is the predicted direction of bias due to missing data? |  | Unpredictable |

| **Bias in measurement of outcomes** | | | |
| --- | --- | --- | --- |
|  | 6.1 Could the outcome measure have been influenced by knowledge of the intervention received? |  | N |
|  | 6.2 Were outcome assessors aware of the intervention received by study participants? |  | N |
|  | 6.3 Were the methods of outcome assessment comparable across intervention groups? |  | Y |
|  | 6.4 Were any systematic errors in measurement of the outcome related to intervention received? |  | N |
|  | **Risk of bias judgement** |  | Low |
|  | Optional: What is the predicted direction of bias due to measurement of outcomes? |  | Unpredictable |

| **Bias in selection of the reported result** | | | |
| --- | --- | --- | --- |
|  | Is the reported effect estimate likely to be selected, on the basis of the results, from... |  |  |
|  | 7.1. ... multiple outcome *measurements* within the outcome domain? |  | N |
|  | 7.2 ... multiple *analyses* of the intervention-outcome relationship? |  | N |
|  | 7.3 ... different *subgroups*? |  | Y |
|  | **Risk of bias judgement** |  | Moderate |
|  | Optional: What is the predicted direction of bias due to selection of the reported result? |  | Unpredictable |

| **Overall bias** | | | |
| --- | --- | --- | --- |
|  | **Risk of bias judgement** | The authors control for any post-intervention variables that could have been affected by the intervention except age. | Moderate |
|  | Optional: What is the overall predicted direction of bias for this outcome? |  | Favours experimental |

**ROBINS-I tool (Stage II): Imanishi^24^, doi: 10.1016/j.ejca.2018.10.003**

**Specify a target randomized trial specific to the study**

| Design | Matched |
| --- | --- |
| Participants | Patients with lung metastasized colorectal cancer who underwent curative resection |
| Experimental intervention | Adjuvant chemotherapy |
| Comparator | Surgery alone |

**Is your aim for this study…?**

| ■ | to assess the effect of *assignment to* intervention |
| --- | --- |
| □ | to assess the effect of *starting and adhering to* intervention |

**Specify the outcome**

| OS, RFS |
| --- |

**Specify the numerical result being assessed**

| OS Adjuvant chemotherapy vs Surgery alone, HR 1.00, 95%CI 0.69-1.50  DFS Adjuvant chemotherapy vs Surgery alone, HR 1.07, 95%CI 0.82-1.39 |
| --- |

**Preliminary consideration of confounders**

| **(i) Confounding domains listed in the review protocol** | | | | |
| --- | --- | --- | --- | --- |
| Confounding domain | Measured variable(s) | Is there evidence that controlling for this variable was unnecessary?* | Is the confounding domain measured validly and reliably by this variable (or these variables)? | OPTIONAL: Is failure to adjust for this variable (alone) expected to favour the experimental intervention or the comparator? |
| Age | Clinical data | No | Yes | Unpredictable |
| Timing of liver metastasis | Clinical data | No | Yes | Unpredictable |
| Preoperative chemotherapy | Clinical data | No | Yes | Unpredictable |
| AC regimen | Clinical data | No | Yes | Unpredictable |

| **(ii) Additional confounding domains relevant to the setting of this particular study, or which the study authors identified as important** | | | | |
| --- | --- | --- | --- | --- |
| Confounding domain | Measured variable(s) | Is there evidence that controlling for this variable was unnecessary?* | Is the confounding domain measured validly and reliably by this variable (or these variables)? | OPTIONAL: Is failure to adjust for this variable (alone) expected to favour the experimental intervention or the comparator? |
| NA |  |  |  |  |

* In the context of a particular study, variables can be demonstrated not to be confounders and so not included in the analysis: (a) if they are not predictive of the outcome; (b) if they are not predictive of intervention; or (c) because adjustment makes no or minimal difference to the estimated effect of the primary parameter. Note that “no statistically significant association” is not the same as “not predictive”.

**Preliminary consideration of co-interventions**

| **(i) Co-interventions listed in the review protocol** | | |
| --- | --- | --- |
| Co-intervention | Is there evidence that controlling for this co-intervention was unnecessary (e.g. because it was not administered)? | Is presence of this co-intervention likely to favour outcomes in the experimental intervention or the comparator |
| NA |  |  |

| **(ii) Additional co-interventions relevant to the setting of this particular study, or which the study authors identified as important** | | |
| --- | --- | --- |
| Co-intervention | Is there evidence that controlling for this co-intervention was unnecessary (e.g. because it was not administered)? | Is presence of this co-intervention likely to favour outcomes in the experimental intervention or the comparator |
| NA |  |  |

## Risk of bias assessment

|  | **Signalling questions** | **Description** | **Response options** |
| --- | --- | --- | --- |
| **Bias due to confounding** | | | |
|  | 1.1 Is there potential for confounding of the effect of intervention in this study?  **If N/PN to 1.1:** the study can be considered to be at low risk of bias due to confounding and no further signalling questions need be considered |  | Y |
|  | **If Y/PY to 1.1**: determine whether there is a need to assess time-varying confounding: |  |  |
|  | 1.2. Was the analysis based on splitting participants’ follow up time according to intervention received?  **If N/PN**, answer questions relating to baseline confounding (1.4 to 1.6)  **If Y/PY**, go to question 1.3. |  | N |
|  | 1.3. Were intervention discontinuations or switches likely to be related to factors that are prognostic for the outcome?  **If N/PN**, answer questions relating to baseline confounding (1.4 to 1.6)  **If Y/PY**, answer questions relating to both baseline and time-varying confounding (1.7 and 1.8) |  | NA |

|  | **Questions relating to baseline confounding only** | | |
| --- | --- | --- | --- |
|  | 1.4. Did the authors use an appropriate analysis method that controlled for all the important confounding domains? | The authors conducted a propensity score matching analysis. But, there remain several confounding factors, including variations in treatment outcomes across facilities and differences in the implementation systems for adjuvant chemotherapy. | N |
|  | 1.5. **If Y/PY to 1.4**: Were confounding domains that were controlled for measured validly and reliably by the variables available in this study? |  | NA |
|  | 1.6. Did the authors control for any post-intervention variables that could have been affected by the intervention? |  | NA |
|  | **Questions relating to baseline and time-varying confounding** | |  |
|  | 1.7. Did the authors use an appropriate analysis method that controlled for all the important confounding domains and for time-varying confounding? |  | NA |
|  | 1.8. **If Y/PY to 1.7**: Were confounding domains that were controlled for measured validly and reliably by the variables available in this study? |  | NA |
|  | **Risk of bias judgement** |  | Moderate |
|  | Optional: What is the predicted direction of bias due to confounding? |  | Favours experimental |

| **Bias in selection of participants into the study** | | | |
| --- | --- | --- | --- |
|  | 2.1. Was selection of participants into the study (or into the analysis) based on participant characteristics observed after the start of intervention?  **If N/PN to 2.1:** go to 2.4 |  | N |
|  | 2.2. **If Y/PY to 2.1**: Were the post-intervention variables that influenced selection likely to be associated with intervention?  2.3 **If Y/PY to 2.2**: Were the post-intervention variables that influenced selection likely to be influenced by the outcome or a cause of the outcome? |  | NA |
|  | 2.4. Do start of follow-up and start of intervention coincide for most participants? |  | Y |
|  | 2.5. **If Y/PY to 2.2 and 2.3, or N/PN to 2.4**: Were adjustment techniques used that are likely to correct for the presence of selection biases? |  | NA |
|  | **Risk of bias judgement** |  | Low |
|  | Optional: What is the predicted direction of bias due to selection of participants into the study? |  | Unpredictable |

| **Bias in classification of interventions** | | | |
| --- | --- | --- | --- |
|  | 3.1 Were intervention groups clearly defined? |  | Y |
|  | 3.2 Was the information used to define intervention groups recorded at the start of the intervention? |  | Y |
|  | 3.3 Could classification of intervention status have been affected by knowledge of the outcome or risk of the outcome? |  | N |
|  | **Risk of bias judgement** |  | Low |
|  | Optional: What is the predicted direction of bias due to classification of interventions? |  | Unpredictable |

| **Bias due to deviations from intended interventions** | | | |
| --- | --- | --- | --- |
|  | **If your aim for this study is to assess the effect of assignment to intervention, answer questions 4.1 and 4.2** | |  |
|  | 4.1. Were there deviations from the intended intervention beyond what would be expected in usual practice? |  | N |
|  | 4.2. **If Y/PY to 4.1**: Were these deviations from intended intervention unbalanced between groups *and* likely to have affected the outcome? |  | NA |
|  | **If your aim for this study is to assess the effect of starting and adhering to intervention, answer questions 4.3 to 4.6** | |  |
|  | 4.3. Were important co-interventions balanced across intervention groups? |  | Y |
|  | 4.4. Was the intervention implemented successfully for most participants? |  | Y |
|  | 4.5. Did study participants adhere to the assigned intervention regimen? |  | Y |
|  | 4.6. **If N/PN to 4.3, 4.4 or 4.5**: Was an appropriate analysis used to estimate the effect of starting and adhering to the intervention? |  | NA |
|  | **Risk of bias judgement** |  | Low |
|  | Optional: What is the predicted direction of bias due to deviations from the intended interventions? |  | Unpredictable |

| **Bias due to missing data** | | | |
| --- | --- | --- | --- |
|  | 5.1 Were outcome data available for all, or nearly all, participants? |  | Y |
|  | 5.2 Were participants excluded due to missing data on intervention status? |  | N |
|  | 5.3 Were participants excluded due to missing data on other variables needed for the analysis? |  | N |
|  | 5.4 **If PN/N to 5.1, or Y/PY to 5.2 or 5.3**: Are the proportion of participants and reasons for missing data similar across interventions? |  | NA |
|  | 5.5 **If PN/N to 5.1, or Y/PY to 5.2 or 5.3**: Is there evidence that results were robust to the presence of missing data? |  | NA |
|  | **Risk of bias judgement** |  | Low |
|  | Optional: What is the predicted direction of bias due to missing data? |  | Unpredictable |

| **Bias in measurement of outcomes** | | | |
| --- | --- | --- | --- |
|  | 6.1 Could the outcome measure have been influenced by knowledge of the intervention received? |  | N |
|  | 6.2 Were outcome assessors aware of the intervention received by study participants? |  | N |
|  | 6.3 Were the methods of outcome assessment comparable across intervention groups? |  | Y |
|  | 6.4 Were any systematic errors in measurement of the outcome related to intervention received? |  | N |
|  | **Risk of bias judgement** |  | Low |
|  | Optional: What is the predicted direction of bias due to measurement of outcomes? |  | Unpredictable |

| **Bias in selection of the reported result** | | | |
| --- | --- | --- | --- |
|  | Is the reported effect estimate likely to be selected, on the basis of the results, from... |  |  |
|  | 7.1. ... multiple outcome *measurements* within the outcome domain? |  | N |
|  | 7.2 ... multiple *analyses* of the intervention-outcome relationship? |  | N |
|  | 7.3 ... different *subgroups*? |  | N |
|  | **Risk of bias judgement** |  | Low |
|  | Optional: What is the predicted direction of bias due to selection of the reported result? |  | Unpredictable |

| **Overall bias** | | | |
| --- | --- | --- | --- |
|  | **Risk of bias judgement** |  | Low |
|  | Optional: What is the overall predicted direction of bias for this outcome? |  | Unpredictable |

**ROBINS-I tool (Stage II): Hansdotter^25^, doi: 10.1002/jso.27188**

**Specify a target randomized trial specific to the study**

| Design | Cohort |
| --- | --- |
| Participants | Patients with lung metastasized colorectal cancer who underwent curative resection |
| Experimental intervention | Adjuvant chemotherapy |
| Comparator | Surgery alone |

**Is your aim for this study…?**

| ■ | to assess the effect of *assignment to* intervention |
| --- | --- |
| □ | to assess the effect of *starting and adhering to* intervention |

**Specify the outcome**

| OS |
| --- |

**Specify the numerical result being assessed**

| OS Adjuvant chemotherapy vs Surgery alone, HR 0.52, 95%CI 0.22-1.20 |
| --- |

**Preliminary consideration of confounders**

| **(i) Confounding domains listed in the review protocol** | | | | |
| --- | --- | --- | --- | --- |
| Confounding domain | Measured variable(s) | Is there evidence that controlling for this variable was unnecessary?* | Is the confounding domain measured validly and reliably by this variable (or these variables)? | OPTIONAL: Is failure to adjust for this variable (alone) expected to favour the experimental intervention or the comparator? |
| Age | Not reported | NA | NA | Unpredictable |
| Timing of liver metastasis | Not reported | NA | NA | Unpredictable |
| Preoperative chemotherapy | Not reported | NA | NA | Unpredictable |
| AC regimen | Not reported | NA | NA | Unpredictable |

| **(ii) Additional confounding domains relevant to the setting of this particular study, or which the study authors identified as important** | | | | |
| --- | --- | --- | --- | --- |
| Confounding domain | Measured variable(s) | Is there evidence that controlling for this variable was unnecessary?* | Is the confounding domain measured validly and reliably by this variable (or these variables)? | OPTIONAL: Is failure to adjust for this variable (alone) expected to favour the experimental intervention or the comparator? |
| NA |  |  |  |  |

* In the context of a particular study, variables can be demonstrated not to be confounders and so not included in the analysis: (a) if they are not predictive of the outcome; (b) if they are not predictive of intervention; or (c) because adjustment makes no or minimal difference to the estimated effect of the primary parameter. Note that “no statistically significant association” is not the same as “not predictive”.

**Preliminary consideration of co-interventions**

| **(i) Co-interventions listed in the review protocol** | | |
| --- | --- | --- |
| Co-intervention | Is there evidence that controlling for this co-intervention was unnecessary (e.g. because it was not administered)? | Is presence of this co-intervention likely to favour outcomes in the experimental intervention or the comparator |
| NA |  |  |

| **(ii) Additional co-interventions relevant to the setting of this particular study, or which the study authors identified as important** | | |
| --- | --- | --- |
| Co-intervention | Is there evidence that controlling for this co-intervention was unnecessary (e.g. because it was not administered)? | Is presence of this co-intervention likely to favour outcomes in the experimental intervention or the comparator |
| NA |  |  |

**Risk of bias assessment**

|  | **Signalling questions** | **Description** | **Response options** |
| --- | --- | --- | --- |
| **Bias due to confounding** | | | |
|  | 1.1 Is there potential for confounding of the effect of intervention in this study?  **If N/PN to 1.1:** the study can be considered to be at low risk of bias due to confounding and no further signalling questions need be considered |  | Y |
|  | **If Y/PY to 1.1**: determine whether there is a need to assess time-varying confounding: |  |  |
|  | 1.2. Was the analysis based on splitting participants’ follow up time according to intervention received?  **If N/PN**, answer questions relating to baseline confounding (1.4 to 1.6)  **If Y/PY**, go to question 1.3. |  | N |
|  | 1.3. Were intervention discontinuations or switches likely to be related to factors that are prognostic for the outcome?  **If N/PN**, answer questions relating to baseline confounding (1.4 to 1.6)  **If Y/PY**, answer questions relating to both baseline and time-varying confounding (1.7 and 1.8) |  | NA |
|  | **Questions relating to baseline confounding only** | | |
|  | 1.4. Did the authors use an appropriate analysis method that controlled for all the important confounding domains? |  | N |
|  | 1.5. **If Y/PY to 1.4**: Were confounding domains that were controlled for measured validly and reliably by the variables available in this study? |  | N |
|  | 1.6. Did the authors control for any post-intervention variables that could have been affected by the intervention? |  | N |
|  | **Questions relating to baseline and time-varying confounding** | |  |
|  | 1.7. Did the authors use an appropriate analysis method that controlled for all the important confounding domains and for time-varying confounding? |  | NA |
|  | 1.8. **If Y/PY to 1.7**: Were confounding domains that were controlled for measured validly and reliably by the variables available in this study? |  | NA |
|  | **Risk of bias judgement** | Patient characteristics are unknown. | Serious |
|  | Optional: What is the predicted direction of bias due to confounding? |  | Unpredictable |

| **Bias in selection of participants into the study** | | | |
| --- | --- | --- | --- |
|  | 2.1. Was selection of participants into the study (or into the analysis) based on participant characteristics observed after the start of intervention?  **If N/PN to 2.1:** go to 2.4 |  | N |
|  | 2.2. **If Y/PY to 2.1**: Were the post-intervention variables that influenced selection likely to be associated with intervention?  2.3 **If Y/PY to 2.2**: Were the post-intervention variables that influenced selection likely to be influenced by the outcome or a cause of the outcome? |  | NA |
|  | 2.4. Do start of follow-up and start of intervention coincide for most participants? |  | Y |
|  | 2.5. **If Y/PY to 2.2 and 2.3, or N/PN to 2.4**: Were adjustment techniques used that are likely to correct for the presence of selection biases? |  | NA |
|  | **Risk of bias judgement** |  | Moderate |
|  | Optional: What is the predicted direction of bias due to selection of participants into the study? |  | Unpredictable |

| **Bias in classification of interventions** | | | |
| --- | --- | --- | --- |
|  | 3.1 Were intervention groups clearly defined? |  | N |
|  | 3.2 Was the information used to define intervention groups recorded at the start of the intervention? |  | Y |
|  | 3.3 Could classification of intervention status have been affected by knowledge of the outcome or risk of the outcome? |  | N |
|  | **Risk of bias judgement** |  | Moderate |
|  | Optional: What is the predicted direction of bias due to classification of interventions? |  | Unpredictable |

| **Bias due to deviations from intended interventions** | | | |
| --- | --- | --- | --- |
|  | **If your aim for this study is to assess the effect of assignment to intervention, answer questions 4.1 and 4.2** | |  |
|  | 4.1. Were there deviations from the intended intervention beyond what would be expected in usual practice? |  | N |
|  | 4.2. **If Y/PY to 4.1**: Were these deviations from intended intervention unbalanced between groups *and* likely to have affected the outcome? |  | NA |
|  | **If your aim for this study is to assess the effect of starting and adhering to intervention, answer questions 4.3 to 4.6** | |  |
|  | 4.3. Were important co-interventions balanced across intervention groups? |  | NI |
|  | 4.4. Was the intervention implemented successfully for most participants? |  | Y |
|  | 4.5. Did study participants adhere to the assigned intervention regimen? |  | PY |
|  | 4.6. **If N/PN to 4.3, 4.4 or 4.5**: Was an appropriate analysis used to estimate the effect of starting and adhering to the intervention? |  | NI |
|  | **Risk of bias judgement** |  | Moderate |
|  | Optional: What is the predicted direction of bias due to deviations from the intended interventions? |  | Unpredictable |

| **Bias due to missing data** | | | |
| --- | --- | --- | --- |
|  | 5.1 Were outcome data available for all, or nearly all, participants? |  | Y |
|  | 5.2 Were participants excluded due to missing data on intervention status? |  | N |
|  | 5.3 Were participants excluded due to missing data on other variables needed for the analysis? |  | N |
|  | 5.4 **If PN/N to 5.1, or Y/PY to 5.2 or 5.3**: Are the proportion of participants and reasons for missing data similar across interventions? |  | NA |
|  | 5.5 **If PN/N to 5.1, or Y/PY to 5.2 or 5.3**: Is there evidence that results were robust to the presence of missing data? |  | NA |
|  | **Risk of bias judgement** |  | Low |
|  | Optional: What is the predicted direction of bias due to missing data? |  | Unpredictable |

| **Bias in measurement of outcomes** | | | |
| --- | --- | --- | --- |
|  | 6.1 Could the outcome measure have been influenced by knowledge of the intervention received? |  | N |
|  | 6.2 Were outcome assessors aware of the intervention received by study participants? |  | N |
|  | 6.3 Were the methods of outcome assessment comparable across intervention groups? |  | N |
|  | 6.4 Were any systematic errors in measurement of the outcome related to intervention received? |  | N |
|  | **Risk of bias judgement** |  | Moderate |
|  | Optional: What is the predicted direction of bias due to measurement of outcomes? |  | Unpredictable |

| **Bias in selection of the reported result** | | | |
| --- | --- | --- | --- |
|  | Is the reported effect estimate likely to be selected, on the basis of the results, from... |  |  |
|  | 7.1. ... multiple outcome *measurements* within the outcome domain? |  | Y |
|  | 7.2 ... multiple *analyses* of the intervention-outcome relationship? |  | Y |
|  | 7.3 ... different *subgroups*? |  | Y |
|  | **Risk of bias judgement** |  | Moderate |
|  | Optional: What is the predicted direction of bias due to selection of the reported result? |  | Unpredictable |

| **Overall bias** | | | |
| --- | --- | --- | --- |
|  | **Risk of bias judgement** | The authors did not control for any post-intervention variables that could have been affected by the intervention. | Serious |
|  | Optional: What is the overall predicted direction of bias for this outcome? |  | Unpredictable |

**Supplemental Table 4.** Grading of Recommendations, Assessment, Development, and Evaluation summary of evidence

| Outcomes | Number of studies | Risk of bias | Inconsistency | Indirectness | Imprecision | Others | Effect HR (95% CI) | Certainty |
| --- | --- | --- | --- | --- | --- | --- | --- | --- |
| Overall survival | 7 | serious | serious | not serious | serious |  | 0,85 (0,69-1.05) | ⊕⊖⊖⊖  Very Low |
| Disease-free survival | 6 | serious | not serious | not serious | not serious |  | 0.81 (0.66-0.99) | ⊕⊕⊕⊖  Moderate |

HR, hazard ratio; CI, confidence interval.
